# Multiple essential functions of *Plasmodium falciparum* actin-1 during malaria blood-stage development

**DOI:** 10.1186/s12915-017-0406-2

**Published:** 2017-08-15

**Authors:** Sujaan Das, Leandro Lemgruber, Chwen L. Tay, Jake Baum, Markus Meissner

**Affiliations:** 1 Wellcome Centre for Molecular Parasitology, Institute of Infection, Immunity & Inflammation, Glasgow Biomedical Research Centre, University of Glasgow, 120 University Place, Glasgow, G12 8TA UK; 2 Department of Life Sciences, Imperial College London, South Kensington, London, SW7 2AZ UK; 3 Faculty of Veterinary Medicine, Ludwig-Maximilians-University Munich, Munich, Germany

**Keywords:** Actin, Cytoskeleton, Invasion, Apicoplast, Cytokinesis, Egress, Merozoite, Apicomplexa, Malaria, *Plasmodium*, *Plasmodium falciparum*, Actomyosin, Conditional gene disruption

## Abstract

**Background:**

The phylum Apicomplexa includes intracellular parasites causing immense global disease burden, the deadliest of them being the human malaria parasite *Plasmodium falciparum*, which invades and replicates within erythrocytes. The cytoskeletal protein actin is well conserved within apicomplexans but divergent from mammalian actins, and was primarily reported to function during host cell invasion. However, novel invasion mechanisms have been described for several apicomplexans, and specific functions of the acto-myosin system are being reinvestigated. Of the two actin genes in *P. falciparum, actin-1* (*pfact1*) is ubiquitously expressed in all life-cycle stages and is thought to be required for erythrocyte invasion, although its functions during parasite development are unknown, and definitive in vivo characterisation during invasion is lacking.

**Results:**

Here we have used a conditional Cre-*lox* system to investigate the functions of PfACT1 during *P. falciparum* blood-stage development and host cell invasion. We demonstrate that PfACT1 is crucially required for segregation of the plastid-like organelle, the apicoplast, and for efficient daughter cell separation during the final stages of cytokinesis. Surprisingly, we observe that egress from the host cell is not an actin-dependent process. Finally, we show that parasites lacking PfACT1 are capable of microneme secretion, attachment and formation of a junction with the erythrocyte, but are incapable of host cell invasion.

**Conclusions:**

This study provides important mechanistic insights into the definitive essential functions of PfACT1 in *P. falciparum*, which are not only of biological interest, but owing to functional divergence from mammalian actins, could also form the basis for the development of novel therapeutics against apicomplexans.

**Electronic supplementary material:**

The online version of this article (doi:10.1186/s12915-017-0406-2) contains supplementary material, which is available to authorized users.

## Background

The phylum Apicomplexa includes many important human pathogens against which no effective vaccines exist and the number of usable drugs remains scarce. Of note are *Plasmodium,* the aetiological agent of malaria, and *Toxoplasma gondii*, an opportunistic pathogen that leads to fatal disease in immunocompromised patients [1]. Malaria causes almost half a million deaths and immeasurable morbidity every year, with most deaths attributable to *Plasmodium falciparum*, the deadliest of the five parasite species capable of infecting humans [2]. The clinical manifestations of malaria are caused by the asexually reproducing haploid blood stages, which invade erythrocytes and establish themselves within a parasitophorous vacuole (PV) within the host cell. Post-invasion, the intraerythrocytic ring stages grow into trophozoites, which divide their nuclei asynchronously by schizogony to form a multinucleated schizont. The mature schizont undergoes a tightly regulated mechanism of egress to break open the PV and the host cell membranes and release daughter merozoites, thus completing a 48-h asexual cycle.

Actin is a central component of the eukaryotic cytoskeleton; actin polymerisation and depolymerisation together with cargo-carrying myosins ‘walking’ on polymerised actin tracks form the basis for many cellular functions such as locomotion, cell shape maintenance, vesicular trafficking, gene regulation, cell division and a plethora of other processes [3, 4]. Apicomplexans share a conserved acto-myosin system, although species-specific differences exist in the repertoire of myosins [5]. The role of the acto-myosin system during the intracellular development of apicomplexan parasites is largely unknown. However, recent studies in *T. gondii* demonstrated a role for the system in maintenance of the plastid-like organelle, the apicoplast, in dense granule motility and in material transport between individual parasites within a parasitophorous vacuole [6–10]. Similarly, in *Plasmodium* spp. intracellular functions of actin have been suggested, such as during endocytosis [11], secretion [12] and antigenic variation [13, 14].

To date, studies on apicomplexan actin have focussed mainly on its suggested central role during parasite motility and host cell invasion: According to the prevalent view, short, highly dynamic actin filaments are formed between the plasma membrane and the inner membrane complex (IMC) of the parasite and are used as tracks by the MyoA motor complex to generate force during these processes [15]. The MyoA motor complex consists of the myosin light chain 1 (MLC1) and gliding associated proteins (GAPs) that anchor this complex between the IMC and the plasma membrane, although, as recently highlighted, the exact organisation of this motor and its role during host cell invasion are still a matter of debate [16, 17]. Most of our understanding of the molecular players during invasion by apicomplexan parasites, in particular the role of actin, comes from research on *Toxoplasma gondii* and *Plasmodium* spp. [18, 19]. According to the current model, surface ligands derived from micronemes are indirectly linked to the actin cytoskeleton and thereby act as force transmitters that are relocated to the posterior of the parasite by the action of the MyoA motor complex. These ligands are subsequently shed by the action of subtilisin-like and rhomboid proteases to release the tight interaction with the substrate [20–23]. Intriguingly, recent reverse genetic studies in *T. gondii* and *Plasmodium* led to the re-evaluation of several components previously assumed to be crucial for motility and invasion, such as rhomboid proteases, which were believed to be essential for shedding [22, 24], and surface ligands such as merozoite surface protein 1 (MSP1) [25], apical membrane antigen 1 (AMA1) [26] and merozoite TRAP-like protein (MTRAP) [27, 28], which were believed to act as attachment factors or force transmitters. In the case of *Toxoplasma*, the acto-myosin system itself could be fully depleted without completely abrogating gliding motility or invasion, necessitating formulation of new and updated models for these processes [29]. A recent study convincingly demonstrated that membrane dynamics at the entry point, regulated by host cell actin, leads to engulfment of *Toxoplasma* in the absence of MyoA [30]. Whilst similar kinetic models have been proposed for *Plasmodium* merozoite invasion into erythrocytes [31, 32], the contribution of host versus parasite actin during erythrocyte invasion is still unclear. Currently the functional characterisation of *P. falciparum* actin relies on inhibitors for F-actin depolymerisation and polymerisation, such as jasplakinolide, latrunculins and cytochalasins [18]. Recent studies, however, question the specificity of inhibitors for apicomplexan actins [29] and demonstrated that latrunculins are not effective against *Toxoplasma* [29] or *Plasmodium* actin [33]. In the case of the widely used inhibitor cytochalasin D, off-target effects have been reported in *Toxoplasma* [19, 29].

Therefore, a reverse genetic functional analysis of *P. falciparum* actin is required to analyse and validate the functions of the protein in detail and to compare it to the diverse functions found in other apicomplexans. Of particular interest in terms of host cell invasion is the question if, similar to *Toxoplasma gondii*, parasite uptake can be facilitated by the host cell once the acto-myosin system of the parasite is completely inactivated [29, 30].

The *P. falciparum* genome encodes two actin genes [34], *actin-1 (pfact1)* and *actin-2 (pfact2)*, with PfACT1 expressed ubiquitously throughout all life-cycle stages, and PfACT2 confined to the mosquito stages and transmittable sexual stages [35]. Like canonical actins, PfACT2 can form long filaments, and disruption of the gene abrogated exflagellation of male gametocytes [35, 36]. In contrast, *pfact1* has thus far not been disrupted by molecular genetic approaches [36], and hence a classical reverse genetic analysis of PfACT1 function has not been possible. In vitro studies have shown that PfACT1 is only capable of forming short filaments [37, 38] and is thought to be the actin responsible for active invasion by merozoites, although definitive in vivo characterisation is lacking. Moreover, the functions of actin dynamics during blood-stage *P. falciparum* development are largely unknown, despite the fact that PfACT1 mRNA is highly upregulated from the onset of nuclear division (http://plasmodb.org/plasmo/app/record/gene/PF3D7_1246200#transcriptomics), indicating functions for the protein during parasite maturation.

Here we used a dimerisable Cre (DiCre)-based genetic system [39] to conditionally disrupt *pfact1* and determine the functions of the protein during intracellular development and host cell invasion. Importantly, our study highlights functional conservation and unique differences between *Toxoplasma* and *Plasmodium* actin and demonstrates that, in contrast to *Toxoplasma, Plasmodium* critically depends on PfACT1 to invade the host cell, indicating that in this case no parasite uptake can occur via host cell-dependent pathways.

## Results

### Conditional disruption of PfACT1 kills parasites within one replication cycle

**Fig. 1:**
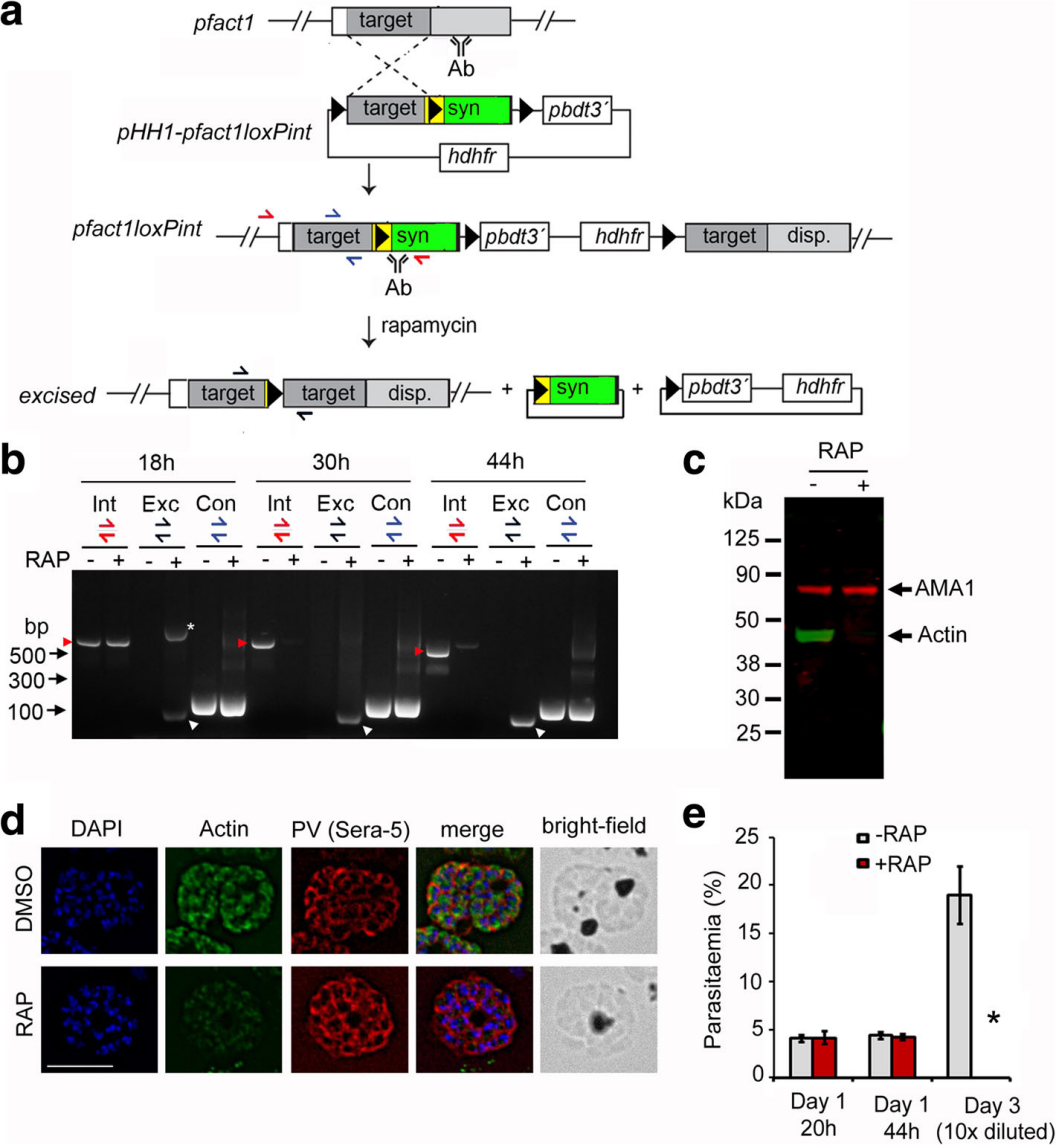
Strategy for modification and conditional disruption of *pfact1*. **a** Schematic showing single crossover allelic replacement of the endogenous *pfact1* gene in the *P. falciparum* 1G5DiCre line with the construct pHH1-pfact1*loxP*int, which includes a 493-bp homologous targeting region (*target*) followed by the *loxP*int module (*yellow*) followed by a recodonised *pfact1* sequence (*syn*), coding for the rest of the *pfact1* open reading frame. *LoxP* sites are depicted as *black triangles*. Integration replaced the *pfact1* 3´ UTR with that of *P. berghei* dihydrofolate reductase gene (*pbdt*). The *hdhfr* cassette confers resistance to the drug WR99210. Following RAP-induced recombination between *loxP* sites, the 3´ half of the *pfact1* gene, coding for the C-terminal 192 aa residues, was removed along with the introduction of STOP codons. Positions of hybridisation of primers for diagnostic polymerase chain reaction (PCR) have been depicted as *red*, *blue* and *black* *half arrows*. **b** Time course of diagnostic PCR with primers showing loss of the integrant *pfact1* locus 18, 34 and 44 h post-RAP treatment (*Int*, *red half arrows* and *red arrow heads*), with a concomitant appearance of the excised locus (*Exc*, *black half arrows* and *white arrow heads*) and no reduction in product of the control PCR (*Con*, *blue half arrows*). The *asterisk* (*) marks an intermediate product of excision which is rapidly lost by 34 h. **c** Western blot showing reactivity to a PfACT1 antibody raised against residues 239 to 253 (*Actin*) is lost within 44 h post-RAP-induced excision; anti-AMA1 staining is used as control. See also Additional file 1: Figure S1b **d** IFA shows a loss of reactivity of PfACT1 KOs to the anti-PfACT1 antibody. The PV is depicted in *red* (Sera-5). Scale bar 5 μm. See also Additional file 2: Figure S2. **e** Parasitaemia counts indicate an abrupt death of PfACT1 KO parasites (RAP) in the following asexual cycle (*day 3*). *N* > 1000. *Error bars* represent standard deviation (*SD*)

We used a DiCre-mediated conditional gene deletion technique [8, 39] to target *pfact1* (Fig. 1a) in *P. falciparum*. In order to not disrupt native actin function, we avoided the use of any epitope tag and employed the strategy of introducing a *loxP* site within a heterologous intron (*loxP*int) in the middle of the *pfact1* gene. Additional *loxP* sites were introduced at the 3´ end of the gene. This approach was previously shown to have no adverse impact on protein expression or function, since the *loxP*int module is efficiently spliced [25, 40]. Furthermore, the promoter region is unaffected by this approach, leading to correct timing of gene expression. The construct pHH1-pfact1*loxP*int, containing a modified *pfact1* genetic sequence with intervening and flanking *loxP* sites, was transfected into the DiCre expressor strain 1G5DiCre [39]. Upon integration into the parasite genome, a line (*loxP*ACT1) was produced in which the C-terminal part of PfACT1 could be efficiently excised upon activation of DiCre with rapamycin (RAP). This effectively resulted in a null mutant, since the 192 amino acid residues in the C-terminal half of the protein are required for actin polymerisation [35]. Two independent clones, B2 and F4, were obtained and used for phenotypic characterisation. Induction of DiCre with pulse treatment of RAP for 4 h in 1 h post-invasion ring stages (Additional file 1: Figure S1a, RAP (at 1 h)) resulted in robust and efficient excision of the target *pfact1* locus (Fig. 1b). The intact locus was excised to completion between 18 and 34 h in the RAP-treated population (Fig. 1b, *red arrow heads*), with protein levels dropping to 13% by 34 h and to <7% by 44 h (Additional file 1: Figure S1b and 1c). PfACT1 disruption was apparent by immunofluorescence assay (IFA) in schizonts 44 h post-induction (Fig. 1d and Additional file 2: Figure S2). Based on IFAs (Additional file 2: Figure S2), it was estimated that ~98% of the population had undergone excision of *pfact1*, resulting in an almost pure population of *pfact1* disrupted parasites (PfACT1 KO) for phenotypic analysis. Only a weak, potentially non-specific signal could be detected by IFA in PfACT1 KO schizonts (Fig. 1d, Additional file 2: Figure S2). We further noted that PfACT1 protein expression in dimethyl sulfoxide (DMSO) controls increased about threefold between 34 h and 44 h as compared to aldolase expression (Additional file 1: Figure S1b), consistent with increased mRNA levels at the late trophozoite stages (http://plasmodb.org/plasmo/app/record/gene/PF3D7_1246200#transcriptomics), indicating functional importance during these stages.

As expected, PfACT1 KOs could not be sustained in culture, formally demonstrating that PfACT1 is indispensable for *P. falciparum* viability (Fig. 1e). No discernible phenotype was observed in Giemsa-stained trophozoite stages (Additional file 1: Figure S1, 26 h, 38 h), but parasites were absent in the following replication cycle (Fig. 1e) with uninvaded merozoites apparent in thin blood films at 48 h post-RAP treatment (Additional file 1: Figure S1, 48 h), implying defects either in mature schizont development, in egress, in erythrocyte invasion or in a combination of these. Interestingly, when excision of PfACT1 was induced by RAP 30 h post-invasion (instead of 1 h post-invasion), the phenotype was consistently apparent at 48 h in the same replication cycle (Additional file 1: Figure S1, RAP (at 30 h)). However, when RAP was added 44 h post-invasion, parasites invaded normally in the same replication cycle but displayed abrogated invasion at the end of the next replication cycle (Additional file 1: Figure S1, RAP (at 44 h)).

### PfACT1 is required for apicoplast segregation and merozoite development

**Fig. 2:**
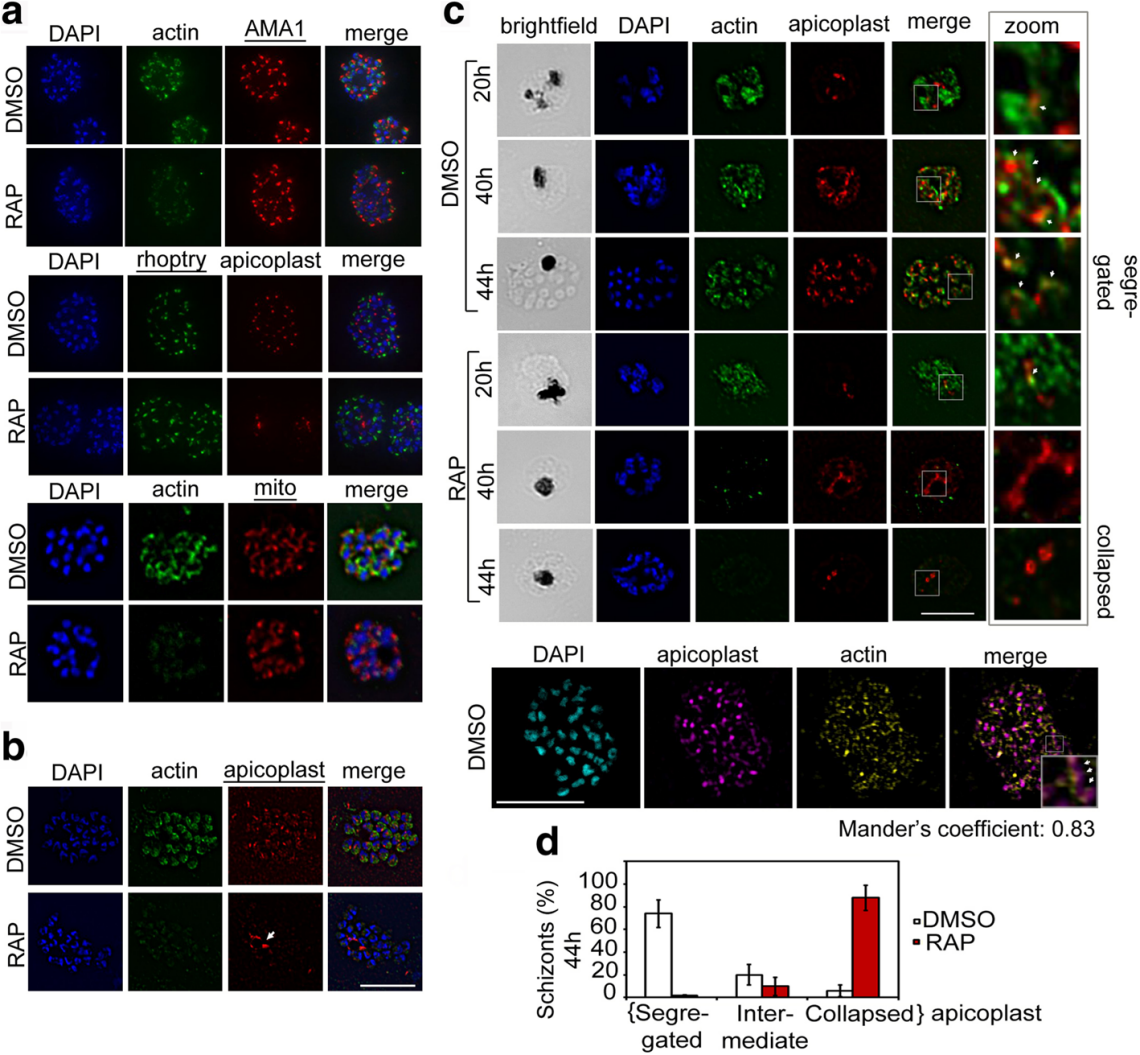
PfACT1 is not required for secretory organelle formation but is crucial for apicoplast segregation. **a** RAP-treated PfACT1 KO parasites have similar microneme (anti-AMA1), rhoptry (anti-RhopH2) and mitochondria (mito; anti-TOM40) architecture as DMSO controls, as revealed by IFA. **b** The apicoplast in PfACT1 KO parasites fails to segregate to daughter merozoites and collapses to an amorphous mass close to the food vacuole (*white arrow*). **c** IFA of samples drawn at various time points shows that apicoplast reticulation and division increase with nuclear division (DMSO, 20, 40, 44 h). The apicoplast shows close apposition to F-actin staining (*zoom*, *white arrows*). Actin staining disappears within 40 h of RAP treatment. The apicoplast does not show reticulation and extensive migration in the absence of PfACT1 (RAP, 40 and 44 h). Scale bar 5 μm. *Bottom panel*: Super-resolution imaging reveals close apposition of apicoplasts on the actin network. Enlarged inset in ‘*merge*’ shows apicoplast colocalised to actin filament (*white arrows*). Colocalisation analysis of apicoplast on actin in the entire image yielded a Manders coefficient of 0.83. **d** Quantification of schizonts showing completely segregated, collapsed or intermediate apicoplasts in DMSO- or RAP-treated populations. *N* > 300.* Error ﻿bars* repre﻿sent *SD.* See also Additional file 1: Figure S1

Maintenance of cellular organelles requires actin in many eukaryotic systems. To identify the role of PfACT1 in organelle biogenesis during intracellular development of the parasite, IFAs on mature segmented schizonts using different organellar markers were performed. No differences in the localisation or structure of the unique secretory organelles in PfACT1 KOs were apparent upon immunostaining (Fig. 2a, top 2 panels). Furthermore, mitochondria architecture in PfACT1 KOs also remained indistinguishable from DMSO controls (Fig. 2a, bottom panel). Strikingly, however, RAP-treated parasites contained a collapsed mass of aberrant apicoplast(s), which presumably had failed to migrate to individual daughter cells (Fig. 2b, white arrow and Additional file 2: Figure S2). In contrast, DMSO controls showed apicoplast staining in each of the daughter merozoites (Fig. 2b and Additional file 2: Figure S2). In order to further investigate apicoplast migration dynamics, we examined apicoplast architecture as a function of time (Fig. 2c). As previously described [41], the apicoplast stained as a simple structure in immature stages (Fig. 2c, DMSO, 20 h) which became complex and reticulated with time (Fig. 2c, DMSO, 40 and 44 h). In mature schizont stages, the apicoplast segregated into individual daughter cells (Fig. 2c, DMSO, 44 h). In contrast, the apicoplast in the PfACT1 KOs showed few branches and eventually collapsed close to the food vacuole (FV) (Fig. 2c, RAP), indicating that apicoplast migration requires actin filaments. At 44 h, >90% of the RAP-treated population showed collapsed apicoplasts (Fig. 2d and Additional file 2: Figure S2). Indeed co-staining with an anti-PfACT1 antibody that preferentially recognises F-actin [42] demonstrated that filamentous F-actin structures connect individual apicoplasts (Fig. 2c, white arrows), which was confirmed by super-resolution microscopy (Fig. 2c, bottom panel). In contrast, similar filaments were never observed in PfACT1 KO parasites, demonstrating specificity of this antibody, as described previously [42]. Together, these results suggest a conserved function of *Plasmodium* and *Toxoplasma* actin-1 [8] in apicoplast segregation.

In order to be eventually passed on from an infected host to a naïve individual, malaria parasites differentiate into male and female gametocytes which are taken up during a blood meal by the female *Anopheles* mosquito. These sexual stages represent a bottleneck in the malaria life cycle, and the development of gametocidal agents holds great promise to combat the spread of the disease [43]. Newly invaded ring stages which will develop into male and female gametocytes are already committed to sexual development in the previous asexual cycle [44]. RAP treatment of newly invaded ring stages in the *loxP*ACT1 clones thus removes the ability of already committed gametocytes to further express PfACT1. We observed that the numbers of gametocytes that persisted 44 h after RAP treatment were comparable to those in the DMSO controls (Additional file 3: Figure S3), indicating that gametocyte survival, at least within the first 48 h, is not reliant on *de novo* expression of PfACT1. Whether these gametocytes will continue developing into more mature forms is beyond the scope of this manuscript and will be the subject of an independent study.

**Fig. 3:**
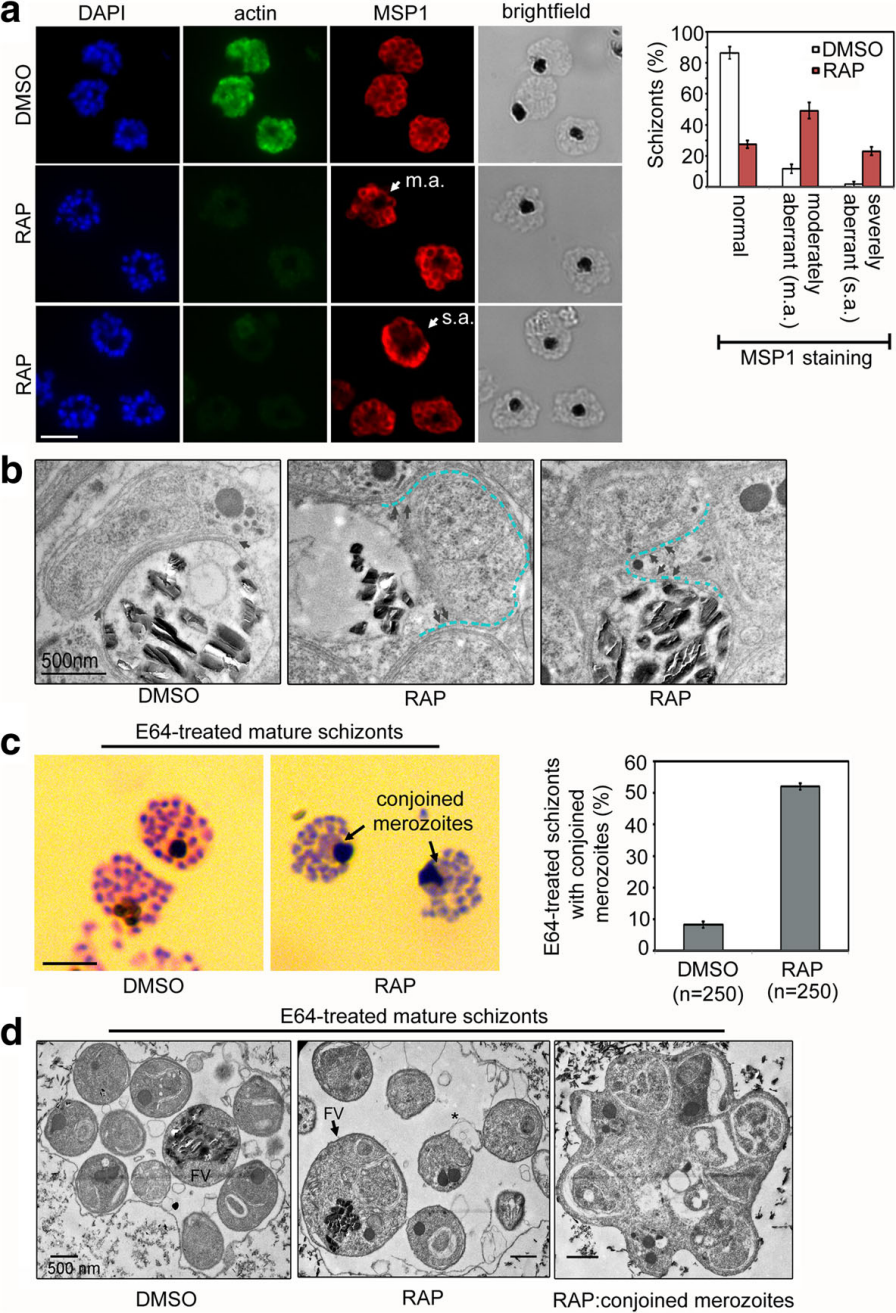
PfACT1 is required for normal cytokinesis. **a** IFA of PfACT1 KO and control schizonts. Aberrant staining of the plasma membrane marker MSP1 (*red*) depicts dysmorphic merozoites in the schizonts (*white arrow*) in the absence of PfACT1 (actin). Scale bar 5 μm. Normal, moderately aberrant (*m.a.*), and severely aberrant (*s.a.*) MSP1 staining has been exemplified (*white arrows*) and quantified (*right*). *N* > 300, *error bars* represent *SD*. **b** TEM on C1-treated mature schizonts stalled just prior to egress. Whilst medially resident daughter merozoites have distinct, separated membranes apposed to the FV membrane in DMSO controls (*black arrows*), aberrant membranous pockets including merozoite material are observed in RAP-treated parasites (*double black arrows and outlined by blue dotted line*). **c** Giemsa-stained, E64-treated mature schizonts show conjoined merozoites (*black arrows*) with ~50% frequency in the RAP-treated sample as compared to <10% in DMSO controls. *N* > 300, scale bar 5 μm. *Error bars* represent *SD*. **d** TEM of E64-trapped merozoites. Merozoites in DMSO controls are distinct and well formed, with organelles not included within the FV membrane (*left panel*). The RAP-treated population has FVs (*black arrow*) which include organelles normally resident in daughter merozoites. Some merozoites show aberrant surface architecture (*asterisked*). Merozoites conjoined to each other are frequently seen in the PfACT1 KO population (*right panel*). TEM scale bar 500 nm. Other scale bars 5 μm

Next, we investigated the role of PfACT1 in daughter cell formation during schizont development. We observed no gross defects in schizont cellular morphology in PfACT1 KO schizonts (Fig. 3a, brightfield). However, aberrant surface staining was observed in RAP-treated schizonts using an anti-MSP1 antibody (Fig. 3a), where the daughter merozoites appeared disorganised and several daughters, particularly in medial regions of the schizont, appeared dysmorphic (Fig. 3a, white arrow). We scored PfACT1 KO schizonts showing normal, moderately aberrant and severely aberrant MSP1 staining (Fig. 3a, m.a. and s.a.). Whilst 86% of DMSO controls showed normal MSP1 staining, 49% of PfACT1 KOs showed moderately aberrant and 23% showed severely aberrant MSP1 staining (Fig. 3a, right panel). Similar results were obtained when we stained for the IMC with an anti-glideosome-associated protein 45 (GAP45) antibody (Additional file 4: Figure S4). In order to further investigate this defect in schizont morphology, we performed transmission electron microscopy (TEM) on PfACT1 KO mature schizonts, cultured for an additional 4–6 h in the presence of the protein kinase G inhibitor Compound 1 (C1), ensuring full development into segmented schizonts, as previously described [45]. In DMSO controls, the boundaries between the daughter merozoite plasma membrane and the FV membrane were well defined (Fig. 3b, DMSO, double black arrows), indicating complete cell separation following segmentation. Surprisingly, RAP-treated parasites showed aberrant membranous pockets adjacent to the FV (Fig. 3b, RAP, double black arrows and blue dotted line), indicating a defect in daughter merozoite formation. Treatment of mature schizonts with the cysteine protease inhibitor E64 traps separated merozoites within a membranous sac [45, 46]. Giemsa staining (Fig. 3c) and TEM (Fig. 3d) on E64-treated preparations of PfACT1 KO parasites unequivocally demonstrated parasite organelles bounded within the same membrane as the FV, strongly suggesting a merozoite formation or cytokinesis defect. Interestingly, a similar role of actin-1 in daughter cell formation has recently been described for *Toxoplasma* [6], indicating conserved function during this process, despite the two genera replicating differently (schizogony and endodyogeny).

To avoid splenic clearance, *P. falciparum*-infected erythrocytes adhere to host vasculature and withdraw from circulation. Adherence is mediated by protrusions called knobs on the infected-erythrocyte surface which contain host cytoskeletal components and parasite-derived proteins [47]. Export of proteins requires trafficking across the parasite plasma membrane and the PV membrane and is thought to be mediated via membranous structures called Maurer’s clefts resident within the host erythrocyte cytoplasm [48]. To assess whether loss of PfACT1 impacts Maurer’s cleft formation, we performed TEM on PfACT1 KO trophozoites and schizonts. RAP-treated parasites were indistinguishable from DMSO controls in their ability to form Maurer’s clefts in the host cytoplasm, suggesting that this process does not rely on parasite actin to proceed (Additional file 5: Figure S5).

### PfACT1 KO merozoites are capable of egress but remain connected to the central FV

Egress from the mature schizont is a well-orchestrated and tightly regulated process in *P. falciparum* development, enabling daughter merozoites to be released in the blood stream to start a new round of invasion and intraerythrocytic growth. When a mature schizont is ready for egress, a key effector serine protease, subtilisin-like protease 1 (SUB1) [49], is released into the PV space in a regulated manner from specialised microneme-like organelles called ‘exonemes’. This is followed by the disruption of the PV membrane and finally the dissemination of daughter merozoites [45]. Actin plays a fundamental role in regulated exocytosis in mammalian cells [50]; it is therefore conceivable that an actin-dependent process may function at the heart of egress, either for secretion of SUB1-containing exonemes or for enabling rapid merozoite movement observed just before host cell rupture [25], especially given that depletion of actin-1 in *Toxoplasma* led to a complete block in host cell egress [7].

**Fig. 4:**
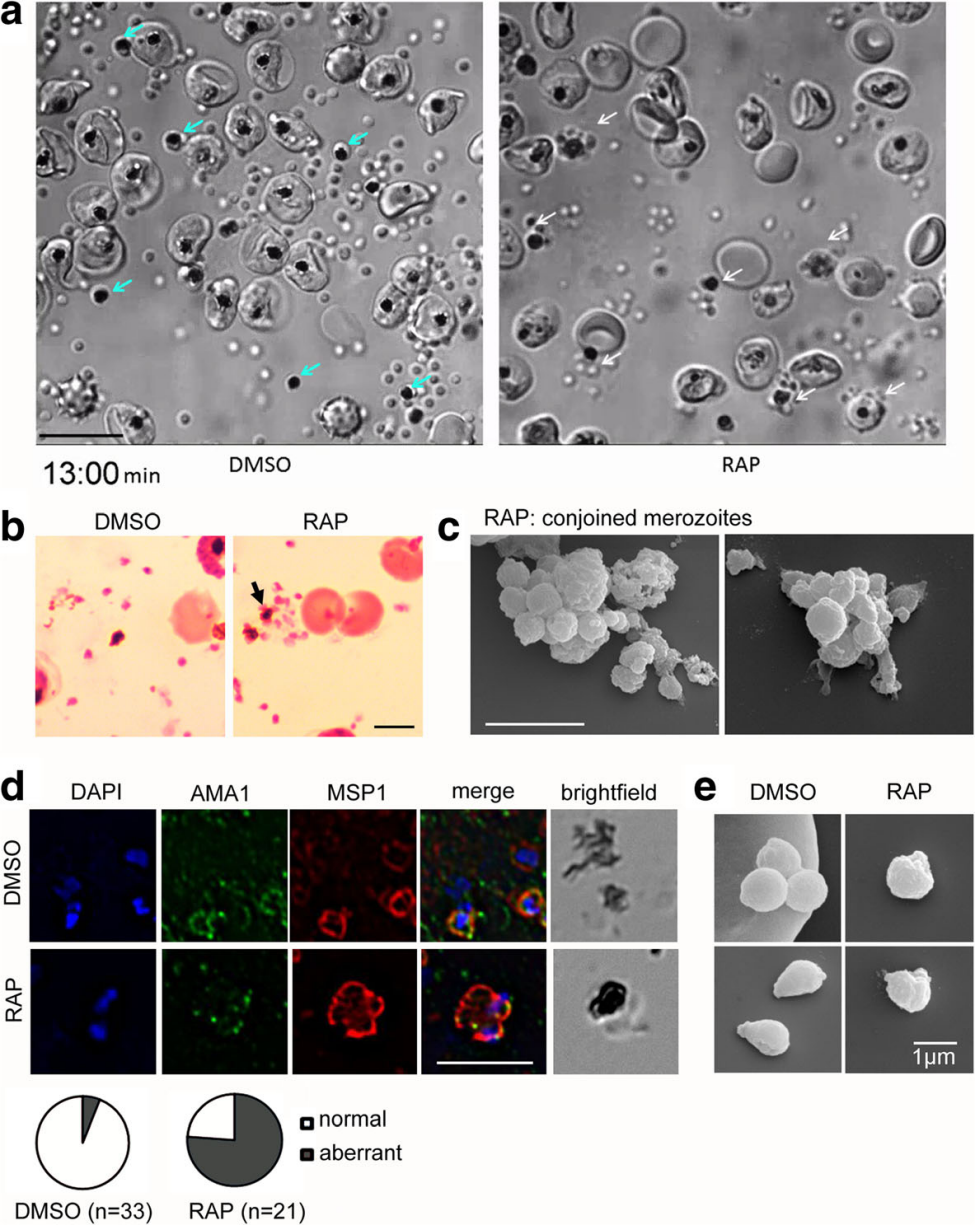
PfACT1 KO merozoites remain conjoined post-egress and possess a dysmorphic ruffled surface. **a** Still images from Additional file 6: Video S1 show normal explosive egress in PfACT1 KOs; however, the RAP-treated population displays conjoined merozoites attached to the FV (*white arrows*), compared to completely segregated merozoites not attached to the FV (*blue arrows*) in DMSO controls. **b** Post-egress, Giemsa-stained RAP-treated populations have parasite structures attached to the FV (*black arrow*). In DMSO controls, the newly released daughter merozoites are free and not connected to the FV. Scale bar 5 μm. **c** Conjoined merozoites with a dysmorphic ruffled surface are apparent by SEM in the RAP-treated population. Scale bar 5 μm. **d** IFA on post-egress preparations of RAP-treated parasites reveals nuclei (*DAPI*) and micronemes (*AMA1*) joined to the FV, with the entire structure bounded by a contiguous plasma membrane (*MSP1*); defect observed in 76% of all FVs. The FV and merozoites are distinct in DMSO controls (94% of all FVs). *N* = 21 for DMSO and *N* = 33 for RAP. Scale bar 5 μm. **e** Free merozoites are released in the PfACT1 KO population, though they often possess a ruffled surface as observed by SEM. Scale bar 1 μm



The reversible protein kinase G inhibitor C1 [51] stalls schizonts at a mature stage of development by blocking the secretion of SUB1. Washing away C1 allows the natural release of merozoites within minutes [45]. We performed time lapse video microscopy of C1-stalled purified PfACT1 KO schizonts to test their ability to complete the asexual cycle and undergo egress. On washing away C1, PfACT1 KO schizonts underwent egress in explosive events similar to the DMSO controls (Additional file 6: Video S1 and Fig. 4a), indicating an actin-independent mechanism of exoneme secretion and egress. However, recapitulating the earlier observed defects in optimal merozoite formation (Fig. 3), a large proportion of the released PfACT1-disrupted merozoites could not separate from each other (Additional file 6: Video S1, RAP, white arrows, and Fig. 4a). In agreement to this, parasite-derived structures in close apposition to the central FV were observed in Giemsa-stained PfACT1 KO populations (Fig. 4b). Scanning electron microscopy (SEM) on the PfACT1 KO population post-egress further showed clusters of conjoined merozoites connected to each other and to the central FV (Fig. 4c), indicating a failure to efficiently separate in the final stages of cytokinesis. On performing an IFA with a merozoite surface marker and a microneme marker, it appeared that these clusters were connected by the parasite plasma membrane and contained nuclei as well as micronemes (Fig. 4d), strongly indicating a partial cytokinesis defect in PfACT1 KO schizonts, as discussed in the previous section.

**Additional file 6: Video S1.** PfACT1 is not required for parasite egress from the host erythrocyte. Time lapse video microscopy of DMSO controls (*left panel*) and RAP-treated PfACT1 KO parasites shows similar mechanics of egress characterised by osmotic burst of merozoites. Unlike the DMSO controls, merozoites connected to the FV and unable to detach are visible in the RAP population (*white arrows*). Relative time shown in minutes, scale bar 10 μm. (MP4 4392 kb)


Despite the presence of non-separated clusters of merozoites, several individual free merozoites were released upon egress in the PfACT1 KO population (Additional file 6: Video S1; Fig. 4a, b, and e). When SEM was performed on these merozoites, we found that, similar to the situation in *T. gondii*, the membrane in PfACT1 KO parasites appeared uneven and ruffled compared to the normally smooth appearance of control parasites (Fig. 4e).

### Loss of PfACT1 leads to a complete abrogation of invasion, despite merozoites retaining the ability to secrete micronemes

**Fig. 5:**
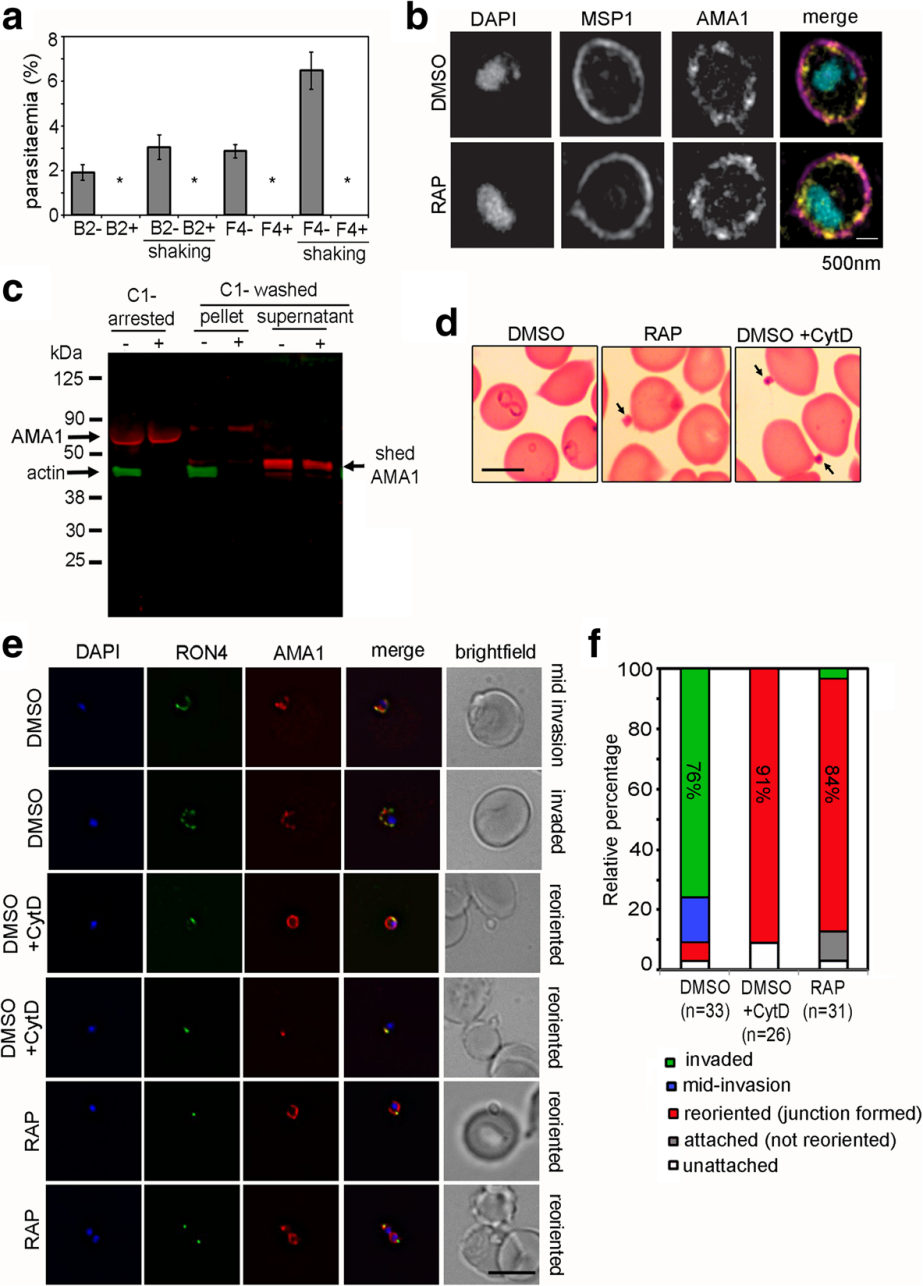
PfACT1 KO merozoites can secrete their micronemes and form a tight junction (*TJ*) but cannot invade erythrocytes. **a** No reinvasion is observed for the RAP-treated population (+) in invasion assays with schizonts purified from two distinct clones B2 and F4. Even upon vigorous shaking, which improved reinvasion rates of the DMSO controls (–), a complete abrogation of invasion was apparent in the RAP-treated population (*). *N* > 1000 in each case. *Error bars* represent *SD*. **b** Super-resolution microscopy of free merozoites. Colocalisation of AMA1 and MSP1 on the surface of PfACT1 KO (*RAP*) and control (*DMSO*) populations indicates that secretion of AMA1 is not ablated in the absence of PfACT1. Scale bar 500 nm. **c** A dual Western blot with anti-AMA1 and anti-PfACT1 antibodies reveals that AMA1 is processed and shed in culture supernatants of the PfACT1 KO population (+), indicating the ability of the PfACT1 KOs to secrete, process and shed AMA1. Note that some AMA1 remains unprocessed in the KOs, perhaps indicative of dysregulation of secretion in a small population of the merozoites. As expected, PfACT1 is absent in the RAP-treated (+) population and is absent in the culture supernatants, confirming integrity of the parasite membranes during the experiment. **d** Merozoites are attached to erythrocytes (*arrowed*) in Giemsa-stained thin blood films from the PfACT1 KO population (*RAP*), which phenocopies cytochalasin-D treatment during invasion (*DMSO + CytD*). DMSO controls reinvade and form ring stages during the time frame of the experiment (*DMSO*). Scale bar 5 μm. **e** IFA of TJ formation. Colocalisation of rhoptry neck protein 4 (*RON4*) and AMA1 at the merozoite-erythrocyte boundary indicates successful TJ formation in DMSO controls (*upper two panels*), controls treated with cytochalasin D (*middle two panels*) and in PfACT1 KOs (*lower two panels*). The state of invasion of the merozoite is depicted on the *right* of the panels (*brightfield*). Scale bar 5 μm. **f** Relative percentages of the state of invasion of merozoites in each of the cases is drawn as a relative bar graph. Each merozoite counted was binned to one of the following groups: invaded, mid-invasion, reoriented, attached (not reoriented) and unattached. The numbers counted have been indicated. Note that the PfACT1 KO (RAP) population closely phenocopies the cytochalasin-D-treated population

We next tested the ability of PfACT1 KO parasites to invade erythrocytes. Two independent clones B2 and F4 showed complete abrogation of invasion upon PfACT1 disruption (Fig. 5a). Since PfACT1 KO parasites are defective in merozoite separation (Fig. 4 and Additional file 6: Video S1), we added a control where the experiment was performed with vigorous shaking of the culture flasks in an attempt to separate any loosely attached merozoites. Despite this, in contrast to controls, the RAP-treated parasites could not invade erythrocytes (Fig. 5a).

Invasion is a multistep process, involving the regulated release of micronemes and rhoptries. Consequently, a blockade in either process could lead to a defect in invasion, and indeed *P. falciparum* actin has been implicated in regulated secretion [12]. We tested the capability of PfACT1 KO parasites to secrete their microneme contents. Super-resolution microscopy revealed that PfACT1 KO parasites retained their ability to secrete the microneme protein AMA1 onto the surface (Fig. 5b). AMA1 is shed from the free merozoite surface by secreted and membrane-resident proteases prior to invasion [20]. We reasoned that if AMA1 is secreted onto the surface, processed AMA1 should be detectable in culture supernatants. Consistent with this, we observed processed AMA1 in the supernatants of egressed PfACT1 KO schizonts by Western blot (Fig. 5c). Moreover, PfACT1 KO free merozoites which were not conjoined displayed no defects in IMC morphology (Additional file 7: Figure S6). Therefore, the abrogation of invasion displayed by PfACT1 KO parasites cannot be attributed to a block in microneme secretion or due to structural inadequacy. In order to qualitatively determine whether the observed lack of invasion by PfACT1 KO parasites is due to the inability of the parasites to attach to erythrocytes, thin blood films were made at the end of the invasion assay. Microscopic observation revealed that PfACT1 KO merozoites could attach to erythrocytes (Fig. 5d, RAP, black arrow). This phenocopied cytochalasin-D-treated control parasites, which could also attach to erythrocytes but could not successfully invade them (Fig. 5d, DMSO + CytD, black arrows).

**Fig. 6:**
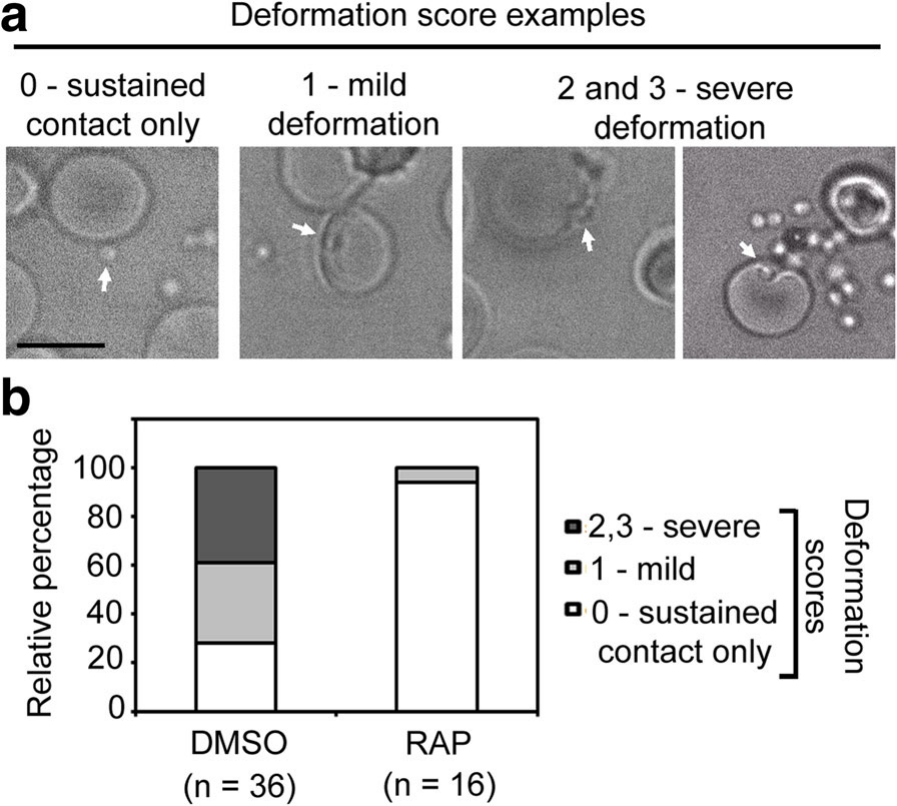
PfACT1 KO merozoites make sustained contacts with erythrocytes but are deficient in their ability to deform them. **a** Still images from time lapse videos showing examples of various degrees of erythrocyte deformation (scored from 0 to 3) caused by merozoites. **b** Quantification of erythrocyte deformation scores of DMSO control and RAP-treated parasites. Numbers counted are depicted *below*

Next, we assessed if attached PfACT1 KO merozoites could form a circular tight junction (TJ), a prerequisite for invasion [52, 53], using the junction markers AMA1 and rhoptry neck protein 4 (RON4) as described previously [54]. Seventy-six percent of DMSO control parasites invaded erythrocytes in the time frame of the assay (Fig. 5e and f). In contrast, 84% of RAP-treated parasites attached to the erythrocyte and could undergo reorientation and appeared to secrete RON proteins which are required for formation of the junction. However, a typical circular junction could never be observed and parasites were incapable of invading erythrocytes, demonstrating a critical requirement for parasite actin for host cell invasion. This observation closely phenocopied cytochalasin-D treatment of the control population (Fig. 5f, DMSO + CytD). Finally, we tested the potency of PfACT1 KO merozoites to deform erythrocytes to which they attached. We performed video microscopy of egressing schizonts in the presence of erythrocytes (Additional file 8: Video S2) and scored the degree of erythrocyte deformation caused by free merozoites as described previously [55]. In DMSO controls, 33% of attached merozoites caused mild deformation (score = 1) and 39% caused severe deformation (score = 2 and 3). In contrast, PfACT1 KO merozoites only formed sustained contacts (score = 0), with only one instance of mild deformation observed (Additional file 8: Video S2 and Fig. 6a, b).

Based on these results, we conclude that PfACT1, in contrast to *Toxoplasma* actin [7], is essential for host cell invasion and that host cell membrane dynamics cannot facilitate uptake of the merozoite in absence of a functional acto-myosin system, as has been described for *T. gondii* [30]. Any parasite in the RAP population that invaded a host cell represented non-excised parasites as evidenced by IFA using an actin antibody (data not shown). Such instances allowed us to confirm the presence of filamentous actin close to the junction in PfACT1-positive merozoites penetrating a host cell (Additional file 9: Figure S7a), as described previously [42]. Cytochalasin -D is known to have off-target effects when used in high concentrations in *Toxoplasma* [29]. Since cytochalasin-D treatment closely phenocopies the invasion phenotypes observed for PfACT1 KO parasites, and in order to determine if the specific target for the drug is indeed PfACT1, we used CRISPR/Cas9 [56] to introduce a single point mutation (Ala136 (GCT) → Gly136 (GGT)) into the *pfact1* genomic locus on an otherwise wild-type 3D7 background (Additional file 9: Figure S7b). Mutation of this site in *Toxoplasma* has previously been shown to confer resistance to cytochalasin [19]. Analysis of three independent cytochalasin-D-resistant mutant clones (Additional file 9: Figure S7c) demonstrated that invasion of erythrocytes was possible in the presence of concentrations of cytochalasin-D where erythrocyte invasion by wild-type parasites is inhibited [57]. This indicates that PfACT1 is indeed the target for erythrocyte invasion inhibition following cytochalasin-D treatment [57] and not an off-target host component. However, higher cytochalasin-D concentrations led to a blockade of invasion in cytochalasin-D-resistant mutants, similar to the situation in *T. gondii* [29]. In fact, cytochalasin-D-resistant mutants could be obtained in *T. gondii,* where no mutation in *actin* could be identified [19].

## Discussion

In this study we analysed the role of PfACT1 during the intraerythrocytic life cycle of *P. falciparum* and discovered highly conserved as well as unique *Plasmodium-*specific functions. Upon induction of DiCre-mediated excision of *pfact1* in 1-h-old ring stages, protein levels of PfACT1 dropped considerably between 24 and 34 h, reaching <7% at 44 h as observed by Western blot (Additional file 1: Figure S1b). Therefore, whilst we have strong evidence for PfACT1 KO phenotypes in late trophozoites, schizonts stages and during invasion, we cannot rule out essential functions of PfACT1 in earlier stages of development, especially in ring stages. Nevertheless, loss of PfACT1 occurred within 40 h in ~98% of RAP-treated parasites (Additional file 2: Figure S2), enabling us to dissect protein function robustly on a population level.

**Fig. 7:**
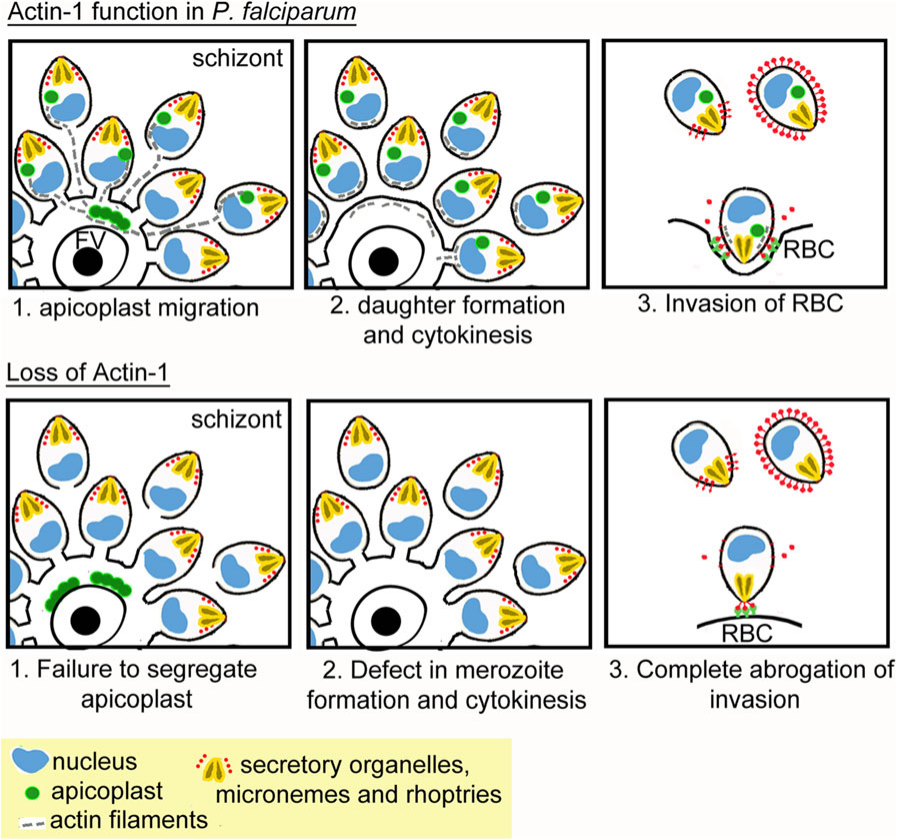
A model visualising the intracellular functions of PfACT1 during *P. falciparum* asexual stages (*upper panels*) and the corresponding phenotypes when PfACT1 function is disrupted (*lower panels*). 1. Apicoplast (*green*) migration requires the presence of PfACT1 filaments (*grey dotted lines*, *upper panel*), failing which they collapse around the FV (*lower panel*). 2. PfACT1 is required to separate daughter cells in the final stages of cytokinesis (*upper panel*). In the absence of PfACT1, conglomerates of merozoites are observed, indicating a role for actin in normal daughter merozoite formation (*lower panel*). 3. Invasion of the host erythrocyte requires the presence of PfACT1 in released daughter merozoites (*upper panel*). In the absence of PfACT1, the merozoite attaches, reorients but is abrogated in its ability to invade the host cell (*lower panel*)

As expected, PfACT1 is essential for parasite viability, and its disruption caused parasite death within one developmental cycle, with phenotypes manifesting at the segmented schizont stage. First, without PfACT1, the apicoplast collapsed and an amorphous mass accumulated close to the FV (Fig. 2, Additional file 2: Figure S2). Although *Toxoplasma* actin is also required for apicoplast maintenance [7], the phenotype is not as drastic as that of *P. falciparum*. We speculate that this difference is due to differences in the mechanism of parasite replication and segregation during endodyogeny (*Toxoplasma*) versus schizogony (*Plasmodium*) and not due to differences in actin function during apicoplast segregation. Intriguingly, individual apicoplasts appear to be connected via filamentous F-actin, as seen in colocalisation analysis using antibodies preferentially recognising F-actin. We speculate that apicoplast replication and inheritance are aided by movement of the apicoplast, potentially along dynamic F-actin structures (Fig. 7). In good agreement, whilst the repertoire of myosins in different apicomplexans is diverse [5], MyoF, which has been demonstrated to be required for apicoplast segregation in *Toxoplasma* [58], is conserved in these parasites, suggesting a highly conserved mechanism.

During schizogony, a defect in merozoite formation/cytokinesis was evident in PfACT1 KOs, especially in medially resident nascent merozoites close to the FV (Fig. 3). Consistent with this, a large proportion of egressed merozoites remained connected to each other in structures resembling bunches of grapes, which contained nuclei as well as apical organelles (Fig. 4). These observations are similar to defects observed in *Toxoplasma* daughter cell formation, which were described as ‘defects in formation of the inner membrane complex’ [6]. The defects in cytokinesis observed are also similar to findings of a recent report where the authors knocked down *P. falciparum* merozoite organising protein (PfMOP) and observed agglomerates of merozoites due to flawed segmentation [59]. Actin-1 in the ciliate *Tetrahymena thermophila*, which is closely related to the apicomplexans, is required for the final stages of cytokinesis during which the daughter cells twist and separate from each other [60]. Specifically, this process was compromised in an *actin-1* knockout, and the cells displayed retraction of the division furrow [60]. We hypothesise that actin-1 may be involved in cell separation during the final stages of cell division in *P. falciparum* (and in *Toxoplasma*) in a similar manner to that in *Tetrahymena* (Fig. 7). In the absence of PfACT1, we speculate that merozoites failed to separate in the very final stages of cytokinesis followed by various degrees of retraction of the cleavage furrow, giving rise to a different number of merozoites conglomerated around the FV in different s﻿chizonts.

Whilst the essential roles of actin during apicoplast segregation and daughter cell separation appear to be highly conserved in apicomplexan parasites, we noted significant differences in actin function in several aspects of parasite development between *Toxoplasma* and *Plasmodium.* Of special interest is the role of actin during host cell egress and invasion. Contrary to our expectations, it appears that egress itself does not require PfACT1 *per se*; data presented in this work (Additional file 6: Video S1) prove that egress in *P. falciparum* does not depend on parasite actin. Consistent with this, treatment of schizonts with cytochalasin-D did not prevent egress of *P. falciparum* schizonts, but completely blocked invasion (Sujaan Das and Michael J Blackman, unpublished, and also evident in Video S8 of another report [55]). Some released PfACT1 KO merozoites were morphologically aberrant, and this is attributable to the defects in cytokinesis discussed above. The force required for egress possibly comes from osmotic swelling and outward curling of the host cell membrane [61] and does not require the function of an acto-myosin motor. In contrast, *Toxoplasma* egress is critically dependent on actin function [7].

In a recent study, *Toxoplasma* parasites were shown to be capable of moving and invading the host cell upon deletion of critical components of the acto-myosin system, including the single-copy *actin* gene [7]*,* demonstrating that *T. gondii* can employ alternative mechanisms for motility and host cell invasion. Indeed it appears that host cells are capable of taking up genetically impaired mutants in a process similar to macropinocytosis [30]. However, based on our results, we can rule out erythrocyte-driven uptake of merozoites as the driving force during invasion, since PfACT1 KO parasites show a complete ablation of entry, demonstrating PfACT1’s essential role in the process.

## Conclusions

In summary, our study demonstrates functional conservation and differences between *Toxoplasma* and *Plasmodium* actin, and we would expect additional differences in function of other core factors of the gliding and invasion machinery. Therefore, whilst cross-comparison between these two species has certainly guided understanding, future comparative studies will be important for defining conserved versus evolutionary niche-specific adaptations of the core molecules required for motility and host cell invasion. Mechanistic dissection of PfACT1 involvement in *P. falciparum* development is not only of biological interest, but owing to the divergence of parasite actin from mammalian actins, can realistically form the basis for development of therapeutics targeting its function towards specific intervention against apicomplexan diseases [62].

## Methods

### Culture and transfection of *P. falciparum*

*P. falciparum* was cultured in RPMI 1640 with Albumax® (Invitrogen), and schizonts were purified on a bed of 70% Percoll® as described previously [63]. About 10 μg of plasmid was ethanol precipitated and resuspended in 10 μL sterile buffer Tris-ethylenediaminetetraacetic acid (EDTA) (Qiagen, Hilden, Germany). The Amaxa™ P3 primary cell 4D-Nucleofector™ X Kit L (Lonza, Cologne, Germany) was used for transfections. The input DNA was added to 100 μL P3 primary cell solution, mixed with 10–20 μL of packed synchronous mature schizonts and added to the cuvette, which was electroporated in a 4D-Nucleofector machine (Lonza) using the program FP158. The transfected schizonts were rapidly added to 2 mL of complete medium (RPMI with Albumax supplemented with glutamine) containing erythrocytes at a haematocrit of 15%, and left shaking in a shaking incubator at 37 °C for 30 min. Finally the cultures were supplemented with 7 mL of complete RPMI medium to obtain a final haematocrit of 3% and incubated overnight at 37 °C in a small angle-necked flask (Nunc™). The presence of the human dihydrofolate reductase (*hdhfr*) selectable marker in the transfection plasmids allowed selection of integrants with the antifolate WR99210 (Jacobus Pharmaceutical Company, Princeton, NJ, USA), added to 2.5 nM 20 h after transfection. The culture medium was subsequently exchanged every day for the next 4 days to remove cell debris which accumulates during electroporation and then twice a week until parasites were detected by Giemsa smear. Drug-resistant parasites were generally detectable in thin blood films 2–3 weeks post-transfection. After this, parasite stocks (at ~5% ring parasitaemia) were cryopreserved in liquid nitrogen, and genomic DNA was prepared for parasites containing integration vectors. Integrants were selected by drug cycling as follows. Drug was removed from the medium and parasites cultured in its absence for 3–4 weeks, after which the drug was added back and the medium changed daily for 2 days. Once parasitaemia was re-established, parasites were cryopreserved in liquid nitrogen, and genomic DNA was prepared using a Qiagen Blood and Tissue kit. The above cycling process was repeated until integration was detectable by PCR analysis. Integration was confirmed by diagnostic PCR using primers UOT_pfact1 _FOR and syn_pfact1_REV. Integrant lines were then cloned by limiting dilution using a simple plaque assay [64], and two clones, B2 and F4, were used for phenotypic characterisation.

### Conditional excision of the *pfact1* locus

Conditional recombination between *loxP* sites was performed as previously described [39]. Briefly, 1-h-old newly invaded ring stages were purified and divided into two flasks. The *pfact1* locus was disrupted by conditional activation of DiCre with a 4-h pulse treatment with 100 nM RAP. The control flask was treated with DMSO for 4 h. The parasites were then washed and returned to culture. Diagnostic PCR was performed at various time points after addition of RAP (18, 34 and 44 h). The intact modified locus was amplified using primers UOT_pfact1_FOR and syn_pfact1_REV (Fig. 1b ‘Int’ and Additional file 10: Table S1), the ‘excised’ locus using primers pfact1_FOR2 and pfact1REV4 (Fig. 1b ‘Exc’ and Additional file 10: Table S1) and the control locus using primers pfact1_FOR1 and pfact1_REV3 (Fig. 1b ‘Con’ and Additional file 10: Table S1). Protein levels were monitored by Western blot as follows. At each time point samples were drawn and erythrocyte membranes disrupted with 0.1% saponin in phosphate-buffered saline (PBS) followed by washes in the same buffer. Parasite protein was subsequently extracted in SDS gel loading buffer supplemented with 100 mM dithiothreitol and boiled for 10 min. We ran 12% polyacrylamide gels, and the proteins were transferred onto a nitrocellulose membrane prior to immunoblotting. Proteins were visualised using the LiCOR Odyssey® imaging system (LiCOR Biosciences, Frankfurt, Germany).

Growth (Fig. 1e) was determined by microscopic counting of parasites from Giemsa-stained thin blood films. On day 2, both the RAP-treated culture and the DMSO control culture were diluted 10× to avoid overgrowth of parasites. Parasitaemia values multiplied by the dilution factor have been presented on the graph (Fig. 1e). At least 1000 erythrocytes were counted at each time point in three independent counts, and the mean parasitaemia values were plotted with standard deviation (SD) as error bars.

### IFA

Thin blood films were made on glass slides and fixed in 4% paraformaldehyde in PBS for 20 min. The slides were then permeabilised with 0.1% Triton-X/PBS for 10 min, washed and blocked overnight in 4% bovine serum albumin (BSA)/PBS. Antigens were labelled with suitable primary (for a list of antibodies used in this study please see Additional file 12: Table S3) and secondary antibodies in 4% BSA/PBS with 5-min PBS washes in between. Slides were finally air dried and mounted with 4’6-diamidino-2-phenylindole (DAPI)-Fluormount-G® (SouthernBiotech, Birmingham, AL, USA).

Staining of the AMA1/RON4 junction in the PfACT1 KOs was performed by fixation and immunostaining in solution as described previously [54]. Along with the comparison of PfACT1 KOs with the DMSO controls, cytochalasin-D treatment of the DMSO population was done at a final concentration of 1 μM. Formation of the junction was quantified as follows. Every parasite was binned in one of the following groups: unattached, attached (not reoriented), reoriented (junction formed), mid-invasion and invaded. Relative percentages of each of the groups were presented in a bar graph.

For image acquisition, z-stacks were collected using a UPLSAPO 100× oil (1.40NA) objective on a Deltavision Core microscope (Image Solutions — Applied Precision, GE) attached to a CoolSNAP HQ2 CCD camera. Deconvolution was performed using SoftWoRx Suite 2.0 (Applied Precision, GE).

An Elyra S1 microscope with Superresolution Structured Illumination (SR-SIM) (Zeiss) was used for super-resolution dissection of AMA1 staining on the merozoite surface and for apicoplast colocalisation with actin filaments.

### Electron microscopy

Samples were fixed in 2.5% glutaraldehyde and 4% paraformaldehyde in 0.1 M phosphate buffer, pH 7.2, for electron microscopy analysis. For TEM, the samples were washed in 0.1 M phosphate buffer, pH 7.2 and post-fixed in 1% OsO_4_ for 1 h on ice. After several washes in the same buffer, the samples were *en bloc* stained with 0.5% uranyl acetate in water for 30 min. Samples were then washed with water, dehydrated in ascending acetone series and resin embedded. Ultrathin sections were collected and imaged on a Tecnai T20 Transmission electron microscope (FEI, Eindhoven, Netherlands).

For SEM, the fixed samples were allowed to adhere on poly-l-lysine-coated coverslips for 20 min, followed by several washes with 0.1 M phosphate buffer. The samples were dehydrated in ascending ethanol series and critical point dried (Tousimis Research, Rockville, MD, USA). The coverslips were mounted on stubs and sputter coated with a 10-nm layer of gold/palladium (Quorum, Laughton, UK) and imaged on a Jeol 6400 scanning electron microscope (Jeol, Tokyo, Japan). All images obtained were analysed and processed with Fiji software [65].

### Time lapse video microscopy of *P. falciparum* parasites

Video microscopy of *P. falciparum* schizont egress was performed as described previously [45]. Synchronised schizonts were Percoll-purified and treated with 2 μM C1 in RPMI medium with Albumax (Gibco) for 4 h. Microscopy chambers (internal volume ~80 μL) for observing live schizonts were constructed by adhering 22 × 64 mm borosilicate glass coverslips to microscope slides with strips of double-sided tape, leaving ~4 mm gaps at each end. C1 was washed off before video microscopy, and the schizonts were immediately resuspended into warm (37 °C) RPMI (with Albumax) and introduced by capillary action into the pre-warmed chamber. The chamber was transferred to a temperature-controlled microscope stage at 37 °C on a Deltavision Core microscope (Image Solutions — Applied Precision, GE). Images were routinely collected at 5-s intervals, beginning 6 min 30 s after washing off C1, over a total of 30 min.

Time lapse video microscopy of erythrocyte invasion by merozoites was performed as described previously [55] with certain modifications. Briefly, synchronised mature schizonts were Percoll-purified and further allowed to mature for 4 h in 2 μM C1. C1 was then washed off, and the schizonts were added to an erythrocyte suspension (0.4% haematocrit) in RPMI medium at 37 °C before transferring to the above-described microscopy chambers. Images were taken every second on a Deltavision Core microscope (Image Solutions — Applied Precision, GE) using a heated stage maintained at 37 °C and 5% CO_2_. Importantly, low light and exposure conditions were maintained to avoid phototoxicity to invading merozoites. Erythrocyte deformation by merozoites was scored as described previously [55] on a scale of 0 to 3 and plotted on a graph. A stringent condition of sustained contact of >1 s was used.

### Invasion and AMA1 shedding assays

Equal numbers of Percoll-purified schizonts from DMSO and RAP-treated cultures were resuspended in RPMI (+Albumax) containing 1% haematocrit blood (volume 4 mL) to a final parasitaemia of ~1–2%. These cultures were further split into two and incubated at 37 °C with or without vigorous shaking for 1 h. Parasitaemia values of newly invaded ring forms were counted by microscopy of Giemsa-stained thin blood films after overnight incubation.

For AMA1 shedding assays, Percoll-purified schizonts from DMSO- and RAP-treated cultures were cultured for an additional 4 h in 2 μM C1 to ensure complete maturation of segmented schizonts. C1 was washed away and the schizonts resuspended in RPMI (without Albumax) and incubated for 30 min at 37 °C. The culture supernatant was separated from the pellet by centrifugation at 13,000 rpm in a benchtop centrifuge. Pellets and supernatants were denatured using reducing SDS sample buffer and used for Western blot using anti-AMA1 and anti-actin antibodies.

### Design of pL7-pfact1AdG

A gene fragment consisting of a recodonised region of the PfACT1 coding sequence (including the C - > G point mutation) and flanked by NT and CT homology regions was synthesised by GeneART and cloned into the pL6-eGFP CRISPR plasmid [56]. The pL6-eGFP plasmid was linearised for cloning using *Sac*II/*Afl*II restriction sites. The guide DNA sequence (ATCCAAAAGGAAATCGTGAG) was cloned into the same plasmid using the *Btg*ZI adaptor site [56], producing the completed pL7-pfact1AdG plasmid. All cloning steps were performed using Gibson assembly [66].

### Invasion assays with cytochalasin-D-resistant mutants

*P. falciparum* schizont stage parasites were diluted to produce a suspension of 2% haematocrit and 2% parasitaemia in media containing increasing concentrations of cytochalasin D (from 0 μM to 0.8 μM). A 10-μL aliquot of the parasite suspension was fixed in 2% paraformaldehyde/0.2% glutaraldehyde/PBS for 45 min at 4 °C. The fixed cells were kept in 50 μL PBS until further use. The remaining parasite suspension was added into a 96-well round-bottomed plate at 100 μL volume per well. After 24 h incubation under standard culture conditions, a 10-μL aliquot of the ring stage parasite suspension was removed from each well and fixed as above. Fixed cells from both days were permeabilised in 0.3% Triton X-100/PBS for 10 min at room temperature and stained with SYBR Green I (Life Technologies)/PBS (1:5000) for 45 min at room temperature in the dark. This was followed by acquisition on a flow cytometer (50,000 events), and parasitaemia was determined by SYBR Green I fluorescence as measured by the flow cytometer. All experiments were carried out in triplicate, and the data are presented as mean ± SD and normalised to the mean parasitaemia of each strain in 0 μM cytochalasin D.

## Additional files

Additional file 1: Figure S1. The phenotypic effect of PfACT1 disruption at various time points in the 48-h development cycle. (**a**) Giemsa-stained thin blood films showing the effect of RAP treatment at various points in the replication cycle. Highly synchronous 1-h-old freshly invaded ring stages were pulse-treated for 4 h with 100 nM rapamycin (RAP at 1 h) or DMSO, washed and returned to culture. Thin blood films were prepared at various time points and Giemsa stained. No phenotype was apparent in the trophozoite stages (26 h, 38 h RAP at 1 h) as compared to DMSO controls, but a complete blockade in invasion was observed in the same replication cycle (48 h, RAP). When RAP treatment was performed at 30 h post-invasion (RAP at 30 h) for 4 h, the phenotypic blockade of invasion was still observed in the same cycle at 48 h. However, when RAP treatment was performed at 44 h post-invasion (RAP at 44 h), invasion occurred normally in the same replication cycle and the phenotypic blockade occurred during invasion in the next replication cycle (48 h, next cycle). Scale bars 5 μm. (**b**) *Left panel*: Western blot showing a time course of loss of PfACT1 (*green*) upon RAP treatment of 1-h-old ring stages, with samples drawn at 24, 34 and 44 h post-induction. Anti-aldolase antibody (*red*) was used as loading control. *Right panel*: Fluorescence intensity of PfACT1 in DMSO controls and RAP-treated population normalised to the intensity of aldolase plotted as a function of time post-RAP treatment. Note that PfACT1 levels in DMSO controls increase about threefold from 34 h to 44 h. (JPEG 2570 kb)

Additional file 2: Figure S2. RAP treatment causes loss of PfACT1 in ~98% of the population together with an apicoplast segregation defect. IFA of parasites harvested at mature schizont stages 44 h post-RAP treatment and further incubated in C1 for 4 h showed loss of PfACT1 in ~98% of the population. A field with one non-excised parasite was deliberately chosen to highlight the specificity of the anti-PfACT1 antibody. Every schizont non-reactive to anti-PfACT1 possessed a collapsed mass of apicoplast(s) evident in the ‘*merge*’ panel. (JPEG 1290 kb)

Additional file 3: Figure S3. Committed gametocytes persist in culture 44 h post-disruption of PfACT1. IFA showing staining of parasites with the gametocyte-specific marker Pfs16. The frequencies of Pfs16-positive parasites in the DMSO controls and in RAP-treated parasites were normalised to the number of schizonts present, and found to be not significantly different from each other (percentages depicted below panel, error intervals represent SD), indicating that sexually committed gametocytes persist 44 h after RAP treatment. Scale bar 5 μm. (JPEG 232 kb)

Additional file 4: Figure S4. IMC formation is aberrant in PfACT1 KO parasites. *Upper panels*: IFA showing GAP45 staining of mature schizonts in DMSO controls or PfACT1 KO population. PfACT1 KO parasites display a disorganised GAP45 staining (*red*), indicating aberrant IMC formation in schizonts. Scale bar 5 μm. *Lower panel*: Quantification of GAP45 staining reveals aberrant IMC formation in ~50–60% of the PfACT1 KO population, *N* > 150. *Error bars* represent *SD*. (JPEG 473 kb)

Additional file 5: Figure S5. Maurer’s cleft formation is not compromised in PfACT1 KO parasites. Representative images of membranous inclusions typical of Maurer’s clefts (*boxed*: 1, 2) are presented. Maurer’s clefts were observed in late trophozoites and schizonts of RAP-treated parasites (*lower two panels*) in 34 of 37 micrographs, and are similar in architecture to DMSO controls (*upper panel*), where they were observed in 25 of 27 micrographs. Boxed regions are presented as larger panels on the *right*. Scale bar 500 nm. (JPEG 2.10 kb)

Additional file 7: Figure S6. Released PfACT1 KO merozoites which are not conjoined do not display any apparent structural defects in the IMC. Representative IFA showing normal IMC staining observed with an anti-MTIP antibody (*red*) in PfACT1 KO parasites. PfACT1 staining is in *green*. Scale bar 1 μm. (JPEG 129 kb)

Additional file 8: Video S2. PfACT1 KO merozoites can attach to erythrocytes but are incompetent in their ability to deform them. Time lapse video microscopy of merozoites egressing from schizonts in the DMSO control population shows significant deformation of erythrocytes (*top panels, blue arrows*), whereas merozoites in the RAP population attach to erythrocytes, but are lacking in their ability to deform them (*lower panels, white arrows*). Relative time shown in minutes, scale bar 5 μm. (MP4 10682 kb) (MP4 10682 kb)

Additional file 9: Figure S7. Targeted mutation of PfACT1 to confer cytochalasin D resistance demonstrates that drug-treated invasion arrest is specific for PfACT1 and not an alternative host factor. (**a**) PfACT1 (*green*) stains the site of junction formation (as marked by *RON4*) during merozoite invasion of the red blood cell. Two merozoites are shown, one in which PfACT1 has been deleted versus a second where PfACT1 is still present. Scale bar 5 μm. (**b**) Genetic strategy for conferring cytochalasin D resistance to the *pfact* gene and PCR confirmation of integration. (**c**) Growth curves of three mutant clones positive for the Ala136 (GCT) → Gly136 (GGT) change, demonstrating that they confer resistance to cytochalasin D compared to a wild-type control. *N* = 50,000. *Error bars* represent *SD*. (JPEG 786 kb)

Additional file 10: Table S1. DNA oligonucleotides used in this study. (DOC 29 kb)

Additional file 11: Table S2. Values for Figure S1b. (XLSX 11 kb)

Additional file 12: Table S3. Antibodies used in this study. (DOC 58 kb)

## Abbreviations

AMA1: apical membrane antigen 1
C1: Compound 1
FV: food vacuole
MSP1: merozoite surface protein 1
PfACT1: *P. falciparum* actin-1
PV: parasitophorous vacuole
RAP: rapamycin
RON2: rhoptry neck protein 2
RON4: rhoptry neck protein 4
SEM: scanning electron microscopy
SUB1: subtilisin-like protease 1
TEM: transmission electron microscopy
TJ: tight junction
