# Supplementary figures and images for: Multiple essential functions of Plasmodium falciparum actin-1 during malaria blood-stage development
Source: BMC Biol. 2017 Aug 15;15:70. doi: 10.1186/s12915-017-0406-2 (PMC5557482; doi:10.1186/s12915-017-0406-2)

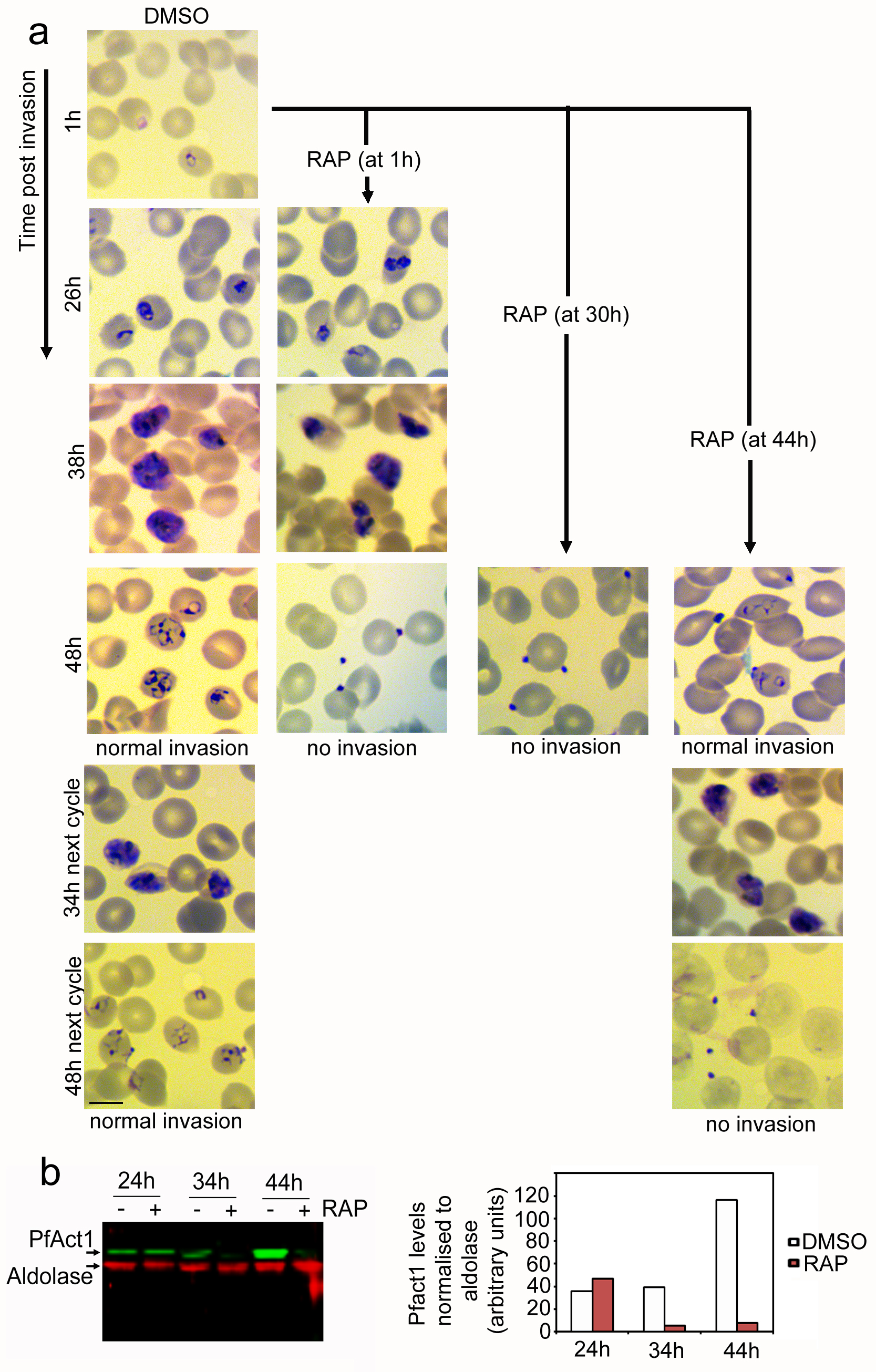

Supplement: Supplementary file 2 — The phenotypic effect of PfACT1 disruption at various time points in the 48-h development cycle. (a) Giemsa-stained thin blood films showing the effect of RAP treatment at various points in the replication cycle. Highly synchronous 1-h-old freshly invaded ring stages were pulse-treated for 4 h with 100 nM rapamycin (RAP at 1 h) or DMSO, washed and returned to culture. Thin blood films were prepared at various time points and Giemsa stained. No phenotype was apparent in the trophozoite stages (26 h, 38 h RAP at 1 h) as compared to DMSO controls, but a complete blockade in invasion was observed in the same replication cycle (48 h, RAP). When RAP treatment was performed at 30 h post-invasion (RAP at 30 h) for 4 h, the phenotypic blockade of invasion was still observed in the same cycle at 48 h. However, when RAP treatment was performed at 44 h post-invasion (RAP at 44 h), invasion occurred normally in the same replication cycle and the phenotypic blockade occurred during invasion in the next replication cycle (48 h, next cycle). Scale bars 5 μm. (b) Left panel: Western blot showing a time course of loss of PfACT1 (green) upon RAP treatment of 1-h-old ring stages, with samples drawn at 24, 34 and 44 h post-induction. Anti-aldolase antibody (red) was used as loading control. Right panel: Fluorescence intensity of PfACT1 in DMSO controls and RAP-treated population normalised to the intensity of aldolase plotted as a function of time post-RAP treatment. Note that PfACT1 levels in DMSO controls increase about threefold from 34 h to 44 h. (JPEG 2570 kb) [file 12915_2017_406_MOESM1_ESM.jpg]

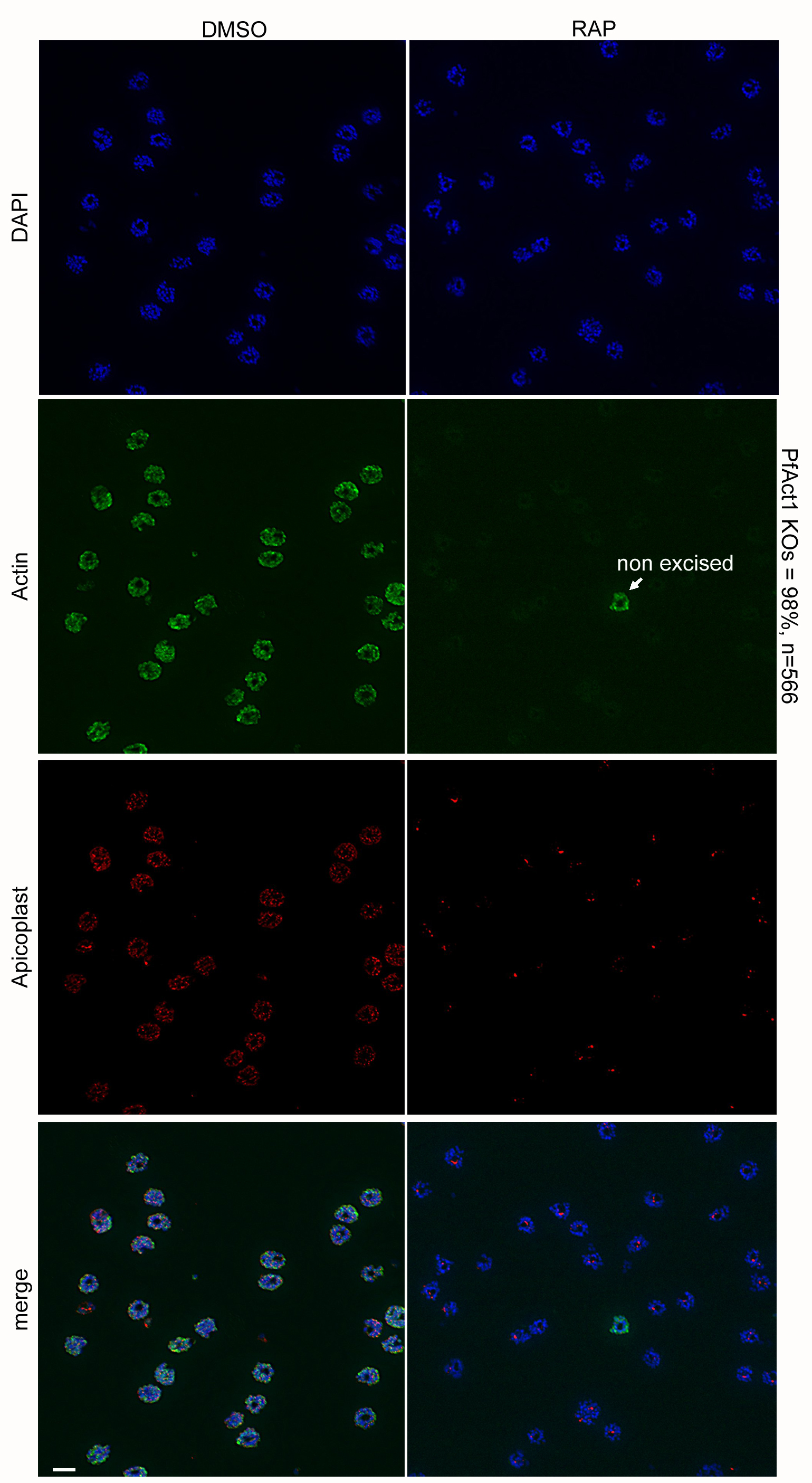

Supplement: Supplementary file 3 — RAP treatment causes loss of PfACT1 in ~98% of the population together with an apicoplast segregation defect. IFA of parasites harvested at mature schizont stages 44 h post-RAP treatment and further incubated in C1 for 4 h showed loss of PfACT1 in ~98% of the population. A field with one non-excised parasite was deliberately chosen to highlight the specificity of the anti-PfACT1 antibody. Every schizont non-reactive to anti-PfACT1 possessed a collapsed mass of apicoplast(s) evident in the ‘merge’ panel. (JPEG 1290 kb) [file 12915_2017_406_MOESM2_ESM.jpg]

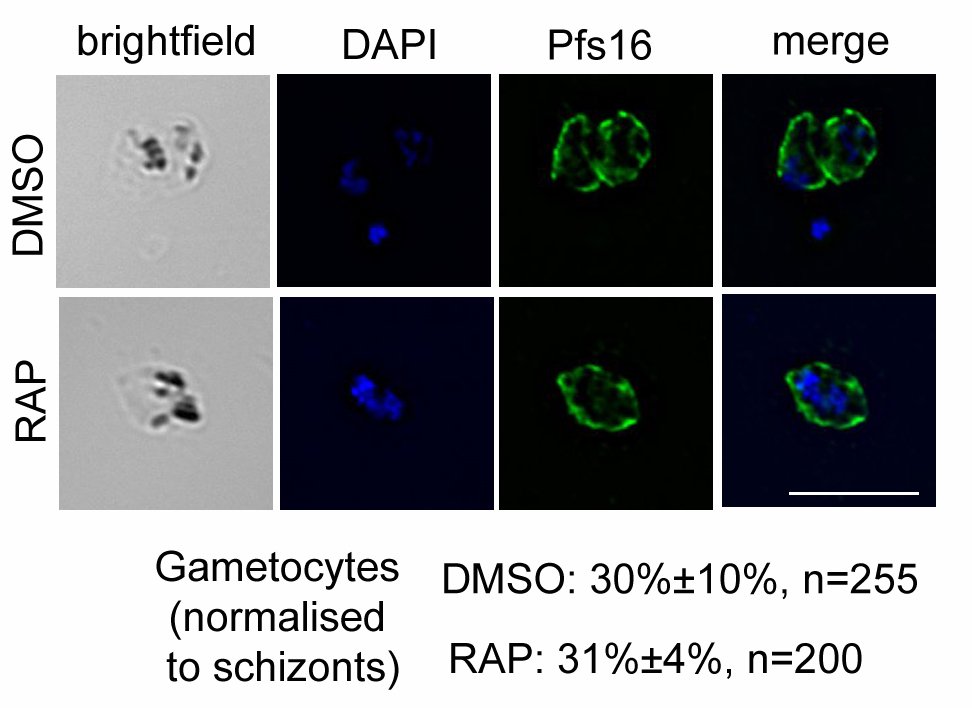

Supplement: Supplementary file 4 — Committed gametocytes persist in culture 44 h post-disruption of PfACT1. IFA showing staining of parasites with the gametocyte-specific marker Pfs16. The frequencies of Pfs16-positive parasites in the DMSO controls and in RAP-treated parasites were normalised to the number of schizonts present, and found to be not significantly different from each other (percentages depicted below panel, error intervals represent SD), indicating that sexually committed gametocytes persist 44 h after RAP treatment. Scale bar 5 μm. (JPEG 232 kb) [file 12915_2017_406_MOESM3_ESM.jpg]

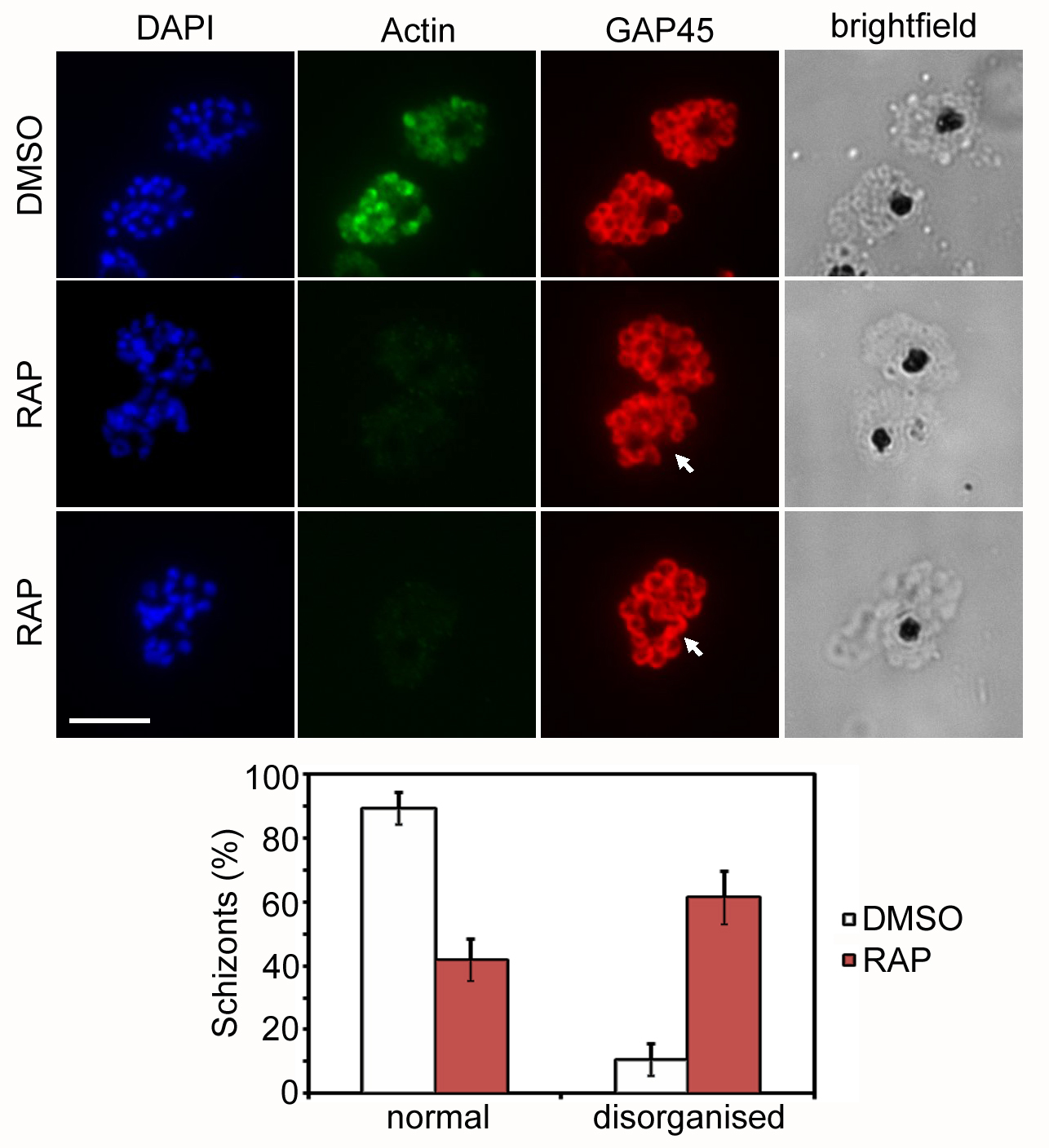

Supplement: Supplementary file 5 — IMC formation is aberrant in PfACT1 KO parasites. Upper panels: IFA showing GAP45 staining of mature schizonts in DMSO controls or PfACT1 KO population. PfACT1 KO parasites display a disorganised GAP45 staining (red), indicating aberrant IMC formation in schizonts. Scale bar 5 μm. Lower panel: Quantification of GAP45 staining reveals aberrant IMC formation in ~50–60% of the PfACT1 KO population, N > 150. Error bars represent SD. (JPEG 473 kb) [file 12915_2017_406_MOESM4_ESM.jpg]

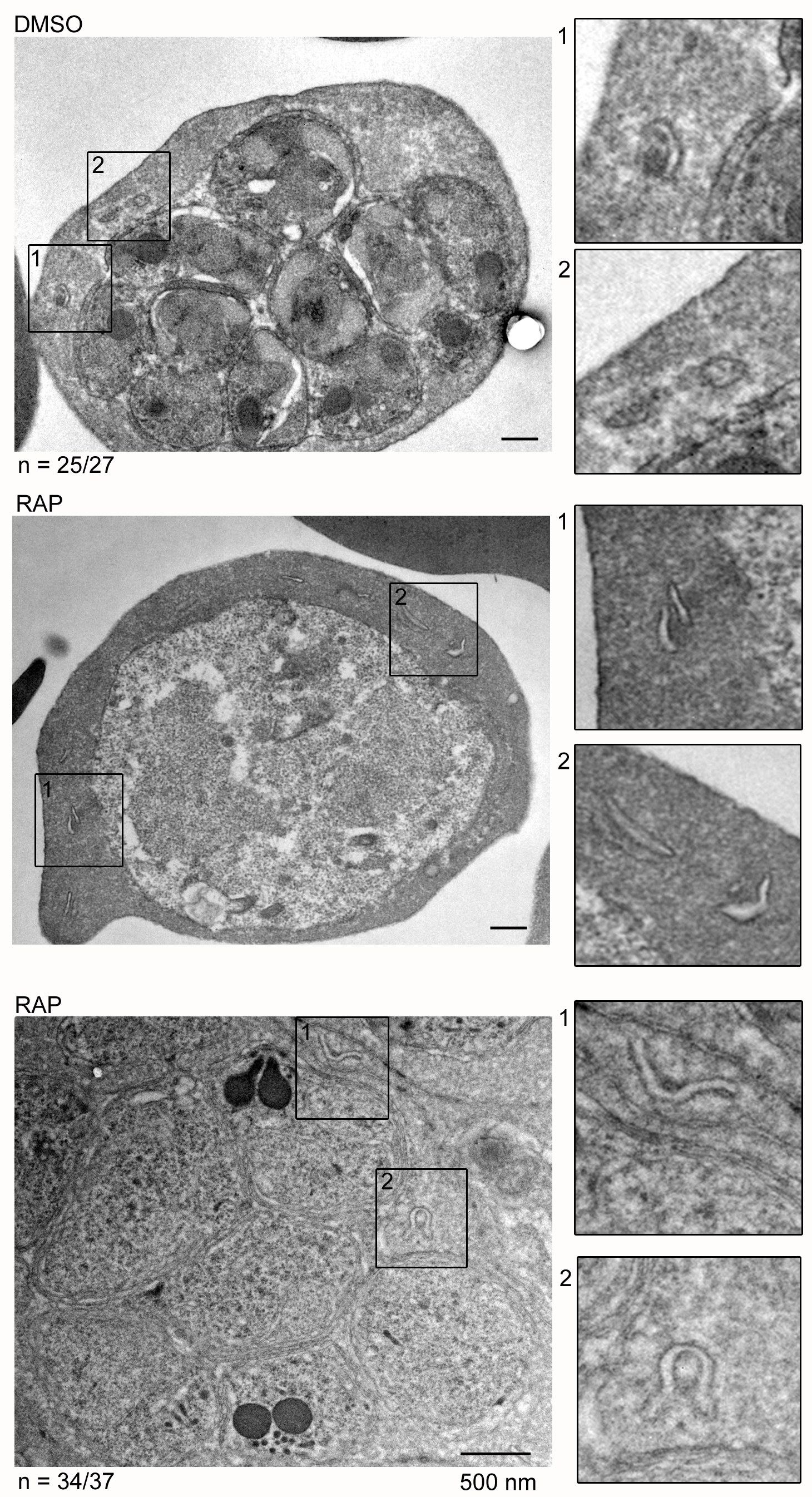

Supplement: Supplementary file 6 — Maurer’s cleft formation is not compromised in PfACT1 KO parasites. Representative images of membranous inclusions typical of Maurer’s clefts (boxed: 1, 2) are presented. Maurer’s clefts were observed in late trophozoites and schizonts of RAP-treated parasites (lower two panels) in 34 of 37 micrographs, and are similar in architecture to DMSO controls (upper panel), where they were observed in 25 of 27 micrographs. Boxed regions are presented as larger panels on the right. Scale bar 500 nm. (JPEG 2.10 kb) [file 12915_2017_406_MOESM5_ESM.jpg]

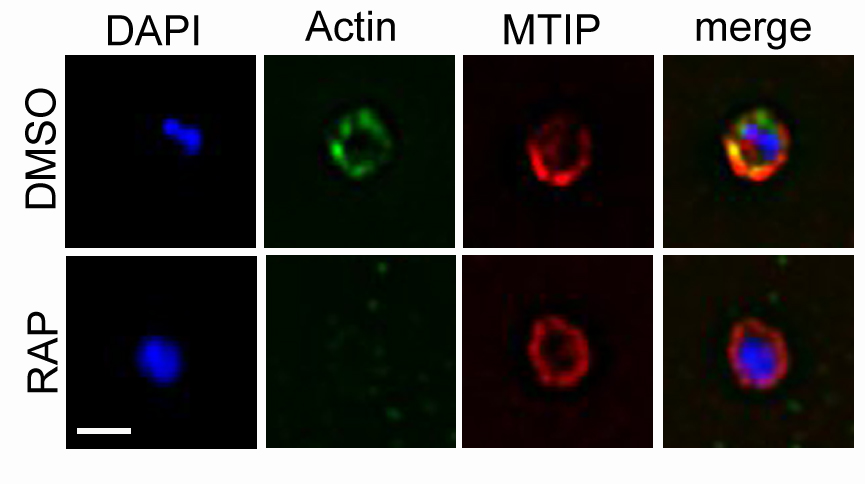

Supplement: Supplementary file 7 — Released PfACT1 KO merozoites which are not conjoined do not display any apparent structural defects in the IMC. Representative IFA showing normal IMC staining observed with an anti-MTIP antibody (red) in PfACT1 KO parasites. PfACT1 staining is in green. Scale bar 1 μm. (JPEG 129 kb) [file 12915_2017_406_MOESM7_ESM.jpg]

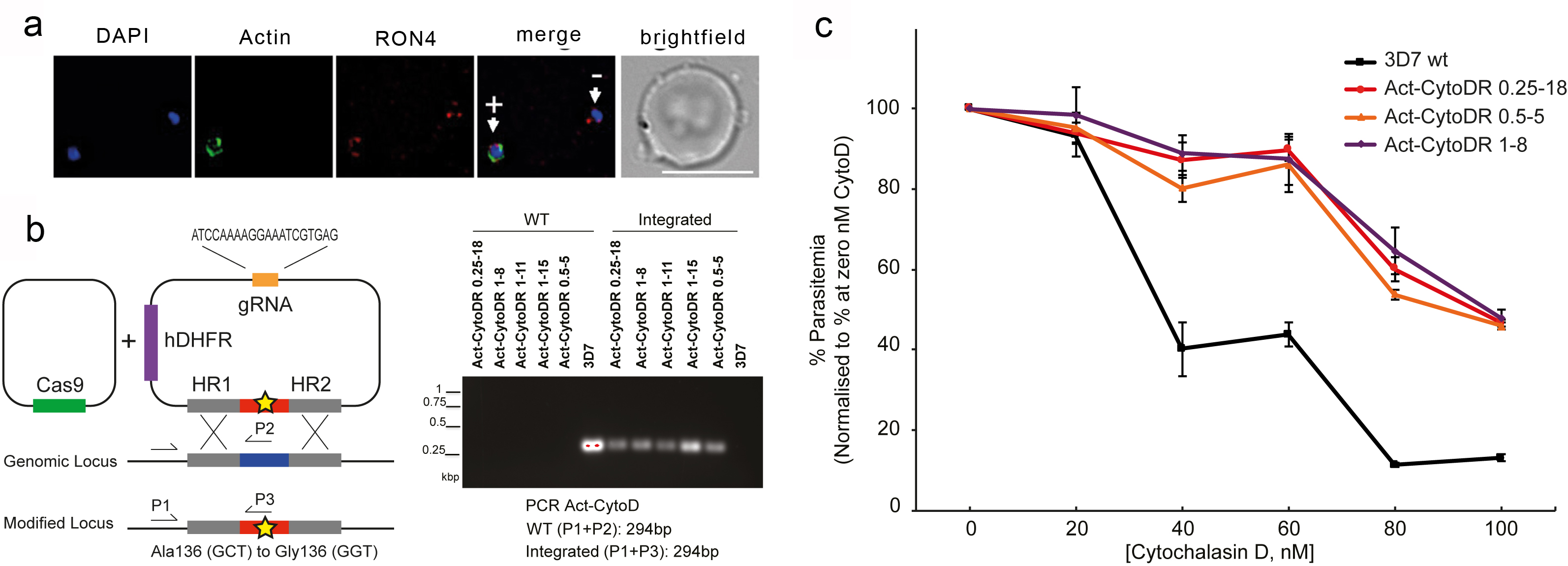

Supplement: Supplementary file 9 — Targeted mutation of PfACT1 to confer cytochalasin D resistance demonstrates that drug-treated invasion arrest is specific for PfACT1 and not an alternative host factor. (a) PfACT1 (green) stains the site of junction formation (as marked by RON4) during merozoite invasion of the red blood cell. Two merozoites are shown, one in which PfACT1 has been deleted versus a second where PfACT1 is still present. Scale bar 5 μm. (b) Genetic strategy for conferring cytochalasin D resistance to the pfact gene and PCR confirmation of integration. (c) Growth curves of three mutant clones positive for the Ala136 (GCT) → Gly136 (GGT) change, demonstrating that they confer resistance to cytochalasin D compared to a wild-type control. N = 50,000. Error bars represent SD. (JPEG 786 kb) [file 12915_2017_406_MOESM9_ESM.jpg]
