# Supplementary material for: Multiple essential functions of Plasmodium falciparum actin-1 during malaria blood-stage development
Source: BMC Biol. 2017 Aug 15;15:70. doi: 10.1186/s12915-017-0406-2 (PMC5557482; doi:10.1186/s12915-017-0406-2)
Supplement: Supplementary file 10 — DNA oligonucleotides used in this study. (DOC 29 kb) [file 12915_2017_406_MOESM10_ESM.doc]

Table S1. DNA oligonucleotides used in this study

| **Primer name** | **Sequence (5´ to 3´)** |
| --- | --- |
| endo_pfact1_REV | CTTCATCAAAATTTAATGC |
| syn_pfact1_REV | CAGCAGAGGTAGAGAAACCG |
| UOT_pfact1_FOR | GTTGTTGACAACGG |
| pfact1_FOR2 | TGAGATTAGATTTAGCTGG |
| Pfact_REV4 | GAACGAGGTGCATCATCTCC |
| pfact1_FOR1 | GCTGCTCCAGAAGAACACCC |
| pfact1_REV3 | GGAACAGTGTGTGATACACC |
