# Supplementary material for: Multiple essential functions of Plasmodium falciparum actin-1 during malaria blood-stage development
Source: BMC Biol. 2017 Aug 15;15:70. doi: 10.1186/s12915-017-0406-2 (PMC5557482; doi:10.1186/s12915-017-0406-2)
Supplement: Supplementary file 12 — Antibodies used in this study. (DOC 58 kb) [file 12915_2017_406_MOESM12_ESM.doc]

**Table S3**. Antibodies used in this study

| **Antibody** | **Reference** |
| --- | --- |
| Mouse anti actin | , RRID: AB_2665920 |
| Mouse anti RhopH2 (61.3) |  |
| Rabbit polyclonal anti MSP1 | Rabbit polyclonal antiserum raised against parasite-derived full-length P. falciparum MSP1 (T9/94 clone) (M. Blackman, unpublished) |
| Rabbit anti CPN60 (apicoplast) |  |
| Rabbit anti TOM40 (mitochondria) |  |
| Mouse anti RON4 |  |
| Rabbit anti AMA1 |  |
| Rabbit anti GAP45 |  |
| Rabbit anti MTIP |  |
| Rabbit anti aldolase |  |
| Mouse anti-Pfs16 | Abmart X2-Q04124 |

References:

67. Holder AA, Freeman RR, Uni S, Aikawa M. Isolation of a Plasmodium falciparum rhoptry protein. Mol Biochem Parasitol. 1985;14(3):293–303.

68. Agrawal S, van Dooren GG, Beatty WL, Striepen B. Genetic evidence that an endosymbiont-derived endoplasmic reticulum-associated protein degradation (ERAD) system functions in import of apicoplast proteins. J Biol Chem. 2009;284(48):33683–91.

69. van Dooren GG, Yeoh LM, Striepen B, McFadden GI. The import of proteins into the mitochondrion of Toxoplasma gondii. J Biol Chem. 2016;291(37): 19335–50.

70. Richard D, MacRaild CA, Riglar DT, Chan JA, Foley M, Baum J, Ralph SA, Norton RS, Cowman AF. Interaction between Plasmodium falciparum apical membrane antigen 1 and the rhoptry neck protein complex defines a key step in the erythrocyte invasion process of malaria parasites. J Biol Chem. 2010;285(19):14815–22.

71. Collins CR, Withers-Martinez C, Hackett F, Blackman MJ. An inhibitory antibody blocks interactions between components of the malarial invasion machinery. PLoS Pathog. 2009;5(1), e1000273.

72. Jones ML, Kitson EL, Rayner JC. Plasmodium falciparum erythrocyte invasion: a conserved myosin associated complex. Mol Biochem Parasitol. 2006; 147(1):74–84.

73. Jewett TJ, Sibley LD. Aldolase forms a bridge between cell surface adhesins and the actin cytoskeleton in apicomplexan parasites. Mol Cell. 2003;11(4): 885–94.
